# The genome of *Nautilus pompilius* illuminates eye evolution and biomineralization

**DOI:** 10.1038/s41559-021-01448-6

**Published:** 2021-05-10

**Authors:** Yang Zhang, Fan Mao, Huawei Mu, Minwei Huang, Yongbo Bao, Lili Wang, Nai-Kei Wong, Shu Xiao, He Dai, Zhiming Xiang, Mingli Ma, Yuanyan Xiong, Ziwei Zhang, Lvping Zhang, Xiaoyuan Song, Fan Wang, Xiyu Mu, Jun Li, Haitao Ma, Yuehuan Zhang, Hongkun Zheng, Oleg Simakov, Ziniu Yu

**Affiliations:** 1 Key Laboratory of Tropical Marine Bio-resources and Ecology and Guangdong Provincial Key Laboratory of Applied Marine Biology, South China Sea Institute of Oceanology, Chinese Academy of Sciences, Guangzhou, China; 2 Innovation Academy of South China Sea Ecology and Environmental Engineering, Chinese Academy of Sciences, Guangzhou, China; 3 Southern Marine Science and Engineering Guangdong Laboratory, Guangzhou, China; 4 MOE Key Laboratory for Membraneless Organelles and Cellular Dynamics, Hefei National Laboratory for Physical Sciences at the Microscale, CAS Key Laboratory of Brain Function and Disease, School of Life Sciences, Division of Life Sciences and Medicine, University of Science and Technology of China, Hefei, China; 5 Zhejiang Key Laboratory of Aquatic Germplasm Resources, College of Biological and Environmental Sciences, Zhejiang Wanli University, Ningbo, China; 6 Biomarker Technologies Corporation, Beijing, China; 7 State Key Laboratory of Biocontrol, College of Life Sciences, Sun Yat-sen University, Guangzhou, China; 8 Department of Neuroscience and Developmental Biology, University of Vienna, Vienna, Austria

**Keywords:** Evolution, Genomics

## Abstract

Nautilus is the sole surviving externally shelled cephalopod from the Palaeozoic. It is unique within cephalopod genealogy and critical to understanding the evolutionary novelties of cephalopods. Here, we present a complete *Nautilus pompilius* genome as a fundamental genomic reference on cephalopod innovations, such as the pinhole eye and biomineralization. Nautilus shows a compact, minimalist genome with few encoding genes and slow evolutionary rates in both non-coding and coding regions among known cephalopods. Importantly, multiple genomic innovations including gene losses, independent contraction and expansion of specific gene families and their associated regulatory networks likely moulded the evolution of the nautilus pinhole eye. The conserved molluscan biomineralization toolkit and lineage-specific repetitive low-complexity domains are essential to the construction of the nautilus shell. The nautilus genome constitutes a valuable resource for reconstructing the evolutionary scenarios and genomic innovations that shape the extant cephalopods.

## Abstract (web summary)

Nautilus, the sole surviving externally shelled cephalopod from the Palaeozoic, holds an important phylogenetic position to understand the evolution of cephalopods. A complete genome of *Nautilus pompilius* sheds light on the evolution of the pinhole eye and biomineralization.

## Main

Nautilus is the only surviving externally shelled cephalopod among hundreds of extinct cephalopod genera since the Palaeozoic; it is deemed unique for its persistent ancestral features despite a long evolutionary history^1^. Palaeobiological evidence shows that the nautilus lineage has preserved plesiomorphic phenotypes such as a chambered shell and primary lens-less eye (pinhole eye)^2^. A phenotypic peculiarity of the adult nautilus shell is that it consists of over 30 chambers: the soft body is accommodated and protected in the outermost chamber, whereas the remaining chambers act as a constant volume hydrostatic apparatus to maintain buoyancy. Moreover, the elegant architecture of the nautilus chambered shell takes the form of a logarithmic spiral conforming to the golden ratio and is composed of sturdy arrays of aragonite crystals, leading to its high degree of hydrostatic stability^3^. Nautilus possesses a unique and simple pinhole eye without lens or cornea, which provides an excellent prototypical model for illuminating the evolution of the eye. Additionally, nautilus is adept in spatial learning and temporally separated biphasic memory even though its brain is disproportionately simple among extant cephalopods^4,5^. As a sister group to nautilus, coleoid cephalopods (such as the octopus, squid and cuttlefish) are perhaps the most intelligent and extraordinarily complex invertebrates with striking morphological and behavioural innovations including sophisticated camera eye, external shell internalization, unusual learning and problem-solving abilities^6–8^. Thus, investigating the nautilus genome could furnish valuable insights into the evolutionary drivers of cephalopod innovations.

Recently, genomic sequencing efforts in coleoids revealed that specific gene family expansions and genome rearrangements may drive the evolution of morphological novelties in these organisms^9–12^. Moreover, transcriptomic analyses have pointed out that RNA editing could allow high plasticity of transcripts, which is associated with thermal adaptation and neural functions^13,14^. However, genomic sequence availability is still limited in coleoid species^9–12^ and a non-coleoid cephalopod genome is urgently needed. In this study, we sequenced the complete genome of *Nautilus pompilius* in the hope of providing a critical reference for the evolution of cephalopods.

*N. pompilius* is the most widespread species among nautiluses and has distributions in the Indo-Pacific region^15^. However, its population has recently declined dramatically due to a mix of unfavourable circumstances, including commercial exploitation of ornamental shells, a lack of legal protection and very slow sexual maturation^16^. Therefore, genome studies of *N. pompilius* would not only shed light on the origin and evolution of cephalopod genomic novelties but also incentivize research on their biology and inform sustainable conservation. Our analyses reveal that the nautilus genome is the smallest when compared to published genomes of coleoid cephalopods; it contains the least number of encoding genes and hitherto the lowest evolutionary rate in the group. Comparative genomics analysis revealed that co-evolution of gene losses and gene family contraction are associated with pinhole eye formation in nautilus, suggesting plausible degeneration from a more complex organ. The unique and new protein-encoding genes in shell formation contribute to the production of aragonite crystals, a major component of the nautilus shell. Moreover, lineage-specific expansion of gene families implicates the active operation of distinct evolutionary strategies of innate immune defence in different cephalopods.

## Results

### Genomic architecture of *N. pompilius*

**Fig. 1:**
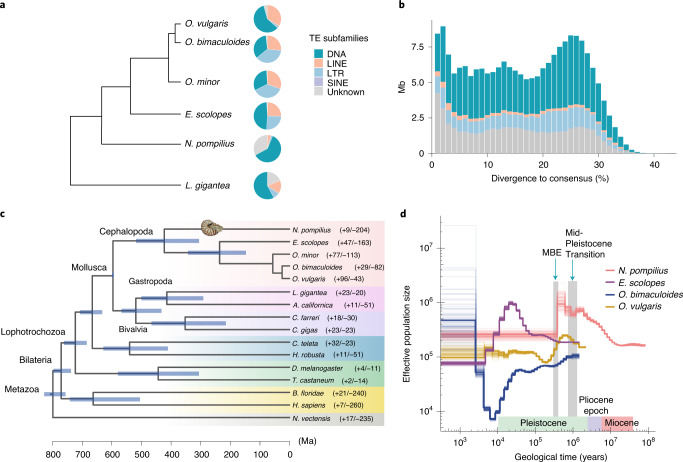
Genomic structure of the *N. pompilius* genome and cephalopod phylogeny. **a**, Proportions of DNA transposons, LTR, LINE and SINE retrotransposons in the genomes of five representative cephalopods including *N. pompilius*, *E. scolopes*, *O. bimaculoides*, *O. minor* and *O. vulgaris*. The tree delineates the evolutionary relationships among the five cephalopod species. The pie charts are scaled according to genome size (Supplementary Fig. 2). **b**, History of TE accumulation in the *N. pompilius* genome. **c**, A phylogenetic tree was constructed with 423 orthologues from 16 metazoan animals using OrthoMCL with a Markov cluster algorithm. Divergence time was estimated with the approximate likelihood calculation method in conjunction with a molecular clock model. A bar within a branch indicates the 95% confidence interval of divergent time. The positive and negative numbers adjacent to the taxon names are gene family numbers of expansion/contraction obtained from the CAFE analysis. **d**, Demographic history of cephalopods. Historical effective population size (Ne) was estimated by using the PSMC method. The synonymous mutation rate per base per year in *N. pompilius* was inferred based on the formula *T* = *ks*/(2*λ*), with a generation time of 15 years. The synonymous mutation rate of *N. pompilius* was estimated as 2.77 × 10^−9^ and that of other cephalopods as 4.07 × 10^−9^. Estimation was performed with 100 bootstraps. Pivotal turning points in environmental evolution during the last million years are labelled with blue arrows.

**Extended Data Fig. 1:**
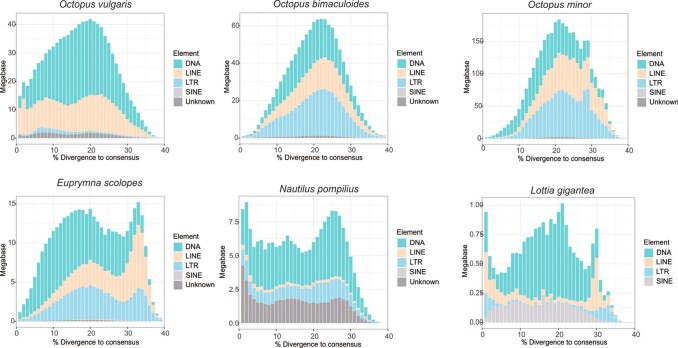
Distribution of the divergence rate of each type of repetitive. Historical transposable element (TE) divergence was compared in the *Octopus bimaculoides*, *Octopus minor*, *Octopus vulgaris*, *Euprymna scolopes*, *Lottia gigantean*, and *Nautilus pompilius*, which were calculated by the Kimura distance-based copy divergence analysis.

The *N. pompiliu*s genome was sequenced with 112.5 coverage of PacBio sequencing reads and 81.8 coverage of Illumina sequencing reads. After de novo assembly via a hybrid approach, these reads were assembled into a 730.58-megabase (Mb) genome with a contig N50 of 1.1 Mb (Supplementary Table 1), which is approximately equal to the estimated genome size of 753.09 Mb by *k*-mer analysis (Supplementary Fig. 1). Integrity of the assembly is demonstrated by 96.83–97.01% of sequencing reads mapping (Supplementary Table 2) and 91.31% of Benchmarking Universal Single-Copy Orthologs (BUSCO) completeness (Supplementary Table 3). The *N. pompiliu*s genome is the smallest among the cephalopods sequenced so far, accounting for only 13.8–41.2% of recently available coleoid genomes (Supplementary Fig. 2)^9–12^. One of the main and ubiquitous genomic components, repetitive elements including transposable elements (TEs), are the driving force in shaping genomic architecture and evolution^17–19^. Comparative analysis further revealed that the make-up of TEs in *N. pompiliu*s is strikingly different to coleoid lineages (Fig. 1a and Supplementary Table 4). In the *N. pompiliu*s genome, TEs make up about 30.95% of the genome where class II DNA transposons predominate (15.55%) whereas class I retrotransposons (long interspersed nuclear element (LINE), long terminal repeat (LTR) and short interspersed nuclear element (SINE)) constitute a minor portion of the genome (6.48%). Retrotransposons were a prominent presence in coleoid cephalopods^9–12^. Furthermore, Kimura distance-based copy divergence analysis indicates that the ancient DNA transposon burst event appeared once; no recent TEs expanded in the *N. pompiliu*s genome (Fig. 1b and Supplementary Fig. 3). In contrast, retrotransposon (LINE and LTR) bursts were observed in coleoid cephalopods (Extended Data Fig. 1 and Table 5), corroborating the critical role of retrotransposons in driving coleoid genome evolution^19^. Therefore, higher proportions of DNA elements and absence of characteristics of retrotransposon expansions make the nautilus genome surprisingly more similar to other molluscan genomes, such as that of *Lottia gigantea*, which is suggestive of slow evolutionary rates in the non-coding regions in nautilus lineages. Moreover, we also examined the evolutionary rates of the coding region in cephalopods based on Tajima’s relative rate test, which revealed slow evolutionary rates in the coding regions of *N. pompilius* (Supplementary Table 6). Consistently, based on the branch lengths of the neutral tree (Supplementary Fig. 4) and actual distances to the out-group (Supplementary Table 7), smaller pairwise distances from *N. pompilius* to *L. gigantea* (4.969 fourfold degenerate (4D) substitutions per site) relative to other coleoid cephalopods to *L. gigantea* (5.132–5.211 4D substitutions per site) were observed. *N. pompilius* apparently experienced fewer intron gains or losses than other coleoid cephalopods after its divergence from the cephalopod ancestor (Supplementary Fig. 4), lending support to its slow-evolving features.

**Extended Data Fig. 2:**
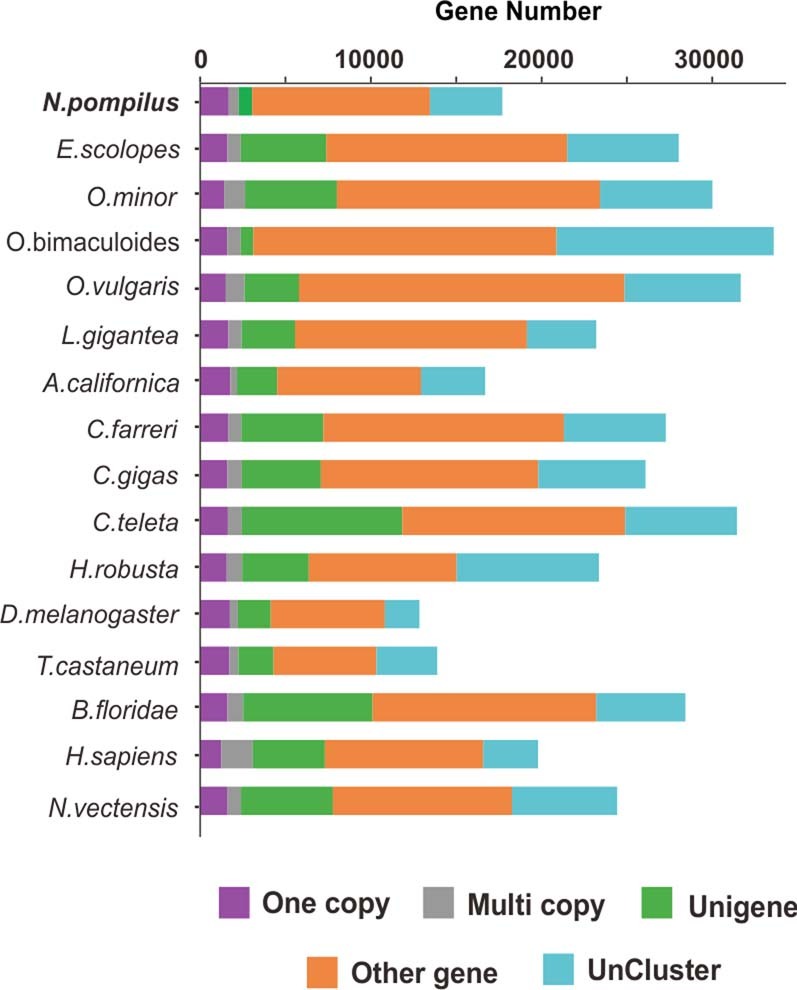
Comparison of gene repertoires in metazoans genomes. ‘One-copy’ indicates single-copy genes. ‘Multi-copy’ indicates orthologous genes present in multiple copies in all taxa. ‘Other gene’ refers to other orthologues that are present in at least one genome. Both ‘Unigene’ and ‘Uncluster’ indicate genes that have not found orthologue in each genome, where ‘Unigene’ contains at least two paralogues. ‘Uncluster’ only contains a single copy.

**Extended Data Fig. 3:**
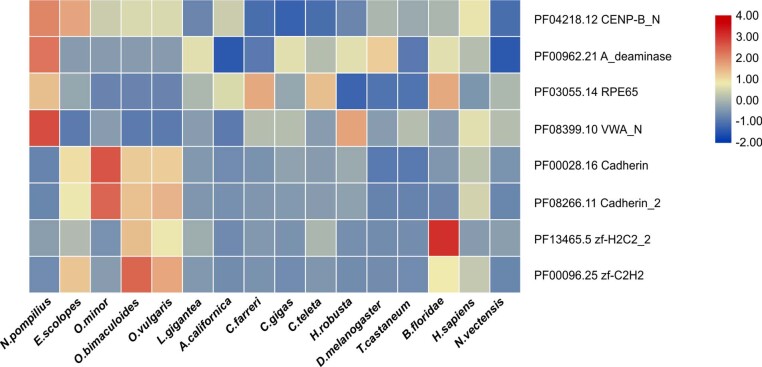
Heatmap on specifically expanded gene families in the *N. pompilius* genome. A number of expanded gene families were found, based on domain analysis in the *N. pompilius* genome. In particular, 18 of the centromere protein B (CENP-B) domain (PF04218.12) containing genes were identified in the *N. pompilius* genome, which makes *N. pompilius* the species with the most CENP-B containing genes in metazoans by far. Also, lineage specific expansion of zinc-finger domains and Cadherin are also observed in the coleoids.

Another cardinal feature of the *N. pompilius* genome is that it encodes relatively fewer genes than the genome of other cephalopods. Whole-genome annotation articulates 17,710 protein-coding genes through integrating multiple methods (Supplementary Fig. 5, Extended Data Fig. 2 and Tables 8 and 9), which is supported by 93.46% BUSCO completeness (Supplementary Table 10). However, this is equivalent to 52.6–60.5% of the gene numbers in octopuses and squids^9–12^. Consistently, Computational Analysis of (gene) Family Evolution (CAFE) analysis reveals a huge contraction of orthologous gene families in the *N. pompilius* genome by the observation of 204 contracted and 9 expanded gene families (Fig. 1c and Supplementary Table 11). Our results also support extensive gene duplications or expansions occurring during coleoid evolution and divergence. Notably, massive expansions of zinc-finger transcription factors and protocadherins, which have previously been noted in the octopus genome with functional implications for neurogenesis and adaptive innovations in the nervous system^9,19^, were not overrepresented in the *N. pompilius* genome (Extended Data Fig. 3). Most strikingly, 18 centromere protein B (CENPB) domain-containing genes were identified and the lineages were specifically expanded in the *N. pompilius* genome (Extended Data Fig. 3). Accumulating evidence has shown that CENPB plays crucial roles in host genome integrity and replication fidelity through the repression of retrotransposons and centromere formation in yeast or humans^20,21^. Therefore, CENPB expansion may serve as a possible host genome surveillance machinery for maintaining integrity of the ancient genome.

### Phylogenetic analysis and population size estimation

To explore the timing and mode of cephalopod evolution, phylogenetic relationships were constructed for 423 single-copy orthologues from 16 animal genomes with OrthoMCL (Fig. 1c). Our phylogenetic results confirm that nautilus is a sister group to coleoids^22^ and their divergence is estimated at around the Silurian–Devonian boundary (422.6 million years ago (Ma)), which is congruent with unequivocal evidence for haemocyanin molecular clock inference (415 Ma) and extensive *Nautilus* fossil records dating back to the early Devonian^23,24^. It was previously hypothesized that diversity of modern coleoid cephalopods emerged during a period of Mesozoic marine revolution^25^. Our results support this assumption in the light of findings on coleoid divergence at the early Triassic (236 Ma), the period after Permian–Triassic extinction^25^. Moreover, our phylogenetic inference further revealed that divergence and speciation of ancient molluscs initiated in the Ediacaran period, during which progressive diversification and biological novelty emerged in the early metazoans^26^.

To better appreciate the dynamic changes in ancestral population sizes of *N. pompilius* and other cephalopods, we assessed the dynamic effective population size (Ne) by employing the pairwise sequential Markovian coalescent (PSMC) method (Fig. 1d). From a perspective of demographic history, profound effects on shaping the *N. pompilius* population are discernible in two crucial environmental evolution events during the last few million years. In particular, *N. pompilius* populations expanded in a stepwise manner at the turn of the Miocene (22.6 Ma). Nevertheless, their ascent came to a halt at the early phase of the Mid-Pleistocene Transition, which is consistent with fundamental climate changes, such as prolongation of glacial cycles prevailing during the period^27^. Most strikingly, a precipitous fall in *N. pompilius* populations occurred at 0.38 Ma, which is close to the onset of the Mid-Brunhes Event (MBE) around 0.4 Ma^28^. The MBE is considered a critical period marked by intensified amplitudes of glacial cycles, wherein variations in ice core temperature and atmospheric CO_2_ concentrations abruptly increased^29,30^. Thus, decimation of the *N. pompilius* population suggests an intrinsic susceptibility to extreme environmental fluctuations. However, we observed that MBE is also a turning point for population expansion of some coleoid species like *Euprymna scolopes* and *Octopus vulgaris*, reflecting the subtle effects of MBE on shaping the demographic composition of cephalopods. Additionally, the effective population size of several bony fishes with a sympatric distribution with nautilus also expanded during the MBE^31,32^, strongly suggesting that ecological competition was likely a pivotal driver of demographic changes in *N. pompilius*.

### Homeobox gene cluster analysis

**Fig. 2:**
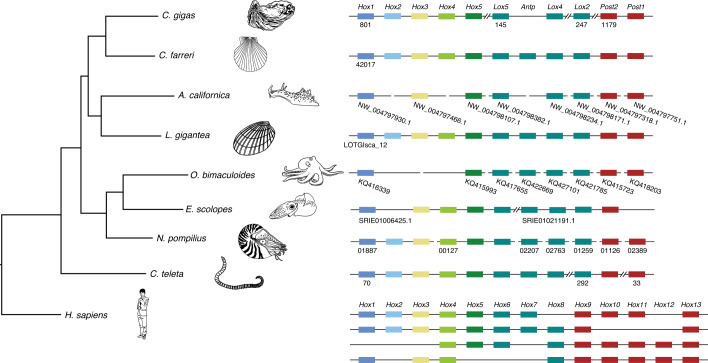
Schematic representation of *Hox* gene clusters in metazoan genomes. Comparison of chromosomal organization of *Hox* gene clusters of *N. pompilius* with other animals. Different *Hox* genes are labelled with coloured boxes. Double slashes indicate that the scaffold of the *Hox* cluster is non-contiguous or interrupted.

Given that homeobox (*Hox*) genes arose as key transcription factors essential to body patterning and tissue segmentation during metazoan evolution^33,34^, it is of great interest to explore the genetic basis for body plan evolution in cephalopods by comparing the organization of *Hox* clusters in multiple lineages. Previous studies have suggested that *Lophotrochozoa* (molluscan) ancestors preserved intact *Hox* clusters^35,36^. In this study, our results show that the *N. pompilius* genome contains a complete set of molluscan *Hox* genes (Fig. 2). Moreover, messenger RNA abundance analysis of *Hox* members reveals a tissue-specific expression patterns in *N. pompilius* (Supplementary Fig. 6). One prominent innovation in coleoids is the loss of an external shell, which has been internalized as a buoyancy compensation apparatus^37^. Consequently, such innovations enabled coleoids to free themselves from a ponderous external shell and drove their remarkable diversification^4^. Correspondingly, *Hox2* in *E. scolopes* and *Hox2*–*Hox4* in *Octopus bimaculoides* are missing (Fig. 2). In parallel, the California sea hare *Aplysia californica*, one of the gastropod species without an external shell, also lost *Hox2*, *Hox4* and *Antp* independently (Fig. 2), suggesting that the disruption of *Hox* cluster integrity may be linked to the evolutionary loss of an external shell in molluscan lineages. Consistent with this view, changes in spatio-temporal collinearity and dorsoventral decoupling of *Hox* gene expression contributed notably to evolutionary diversity in molluscan lineages^35,38^.

### Evolution of the pinhole eye

**Fig. 3:**
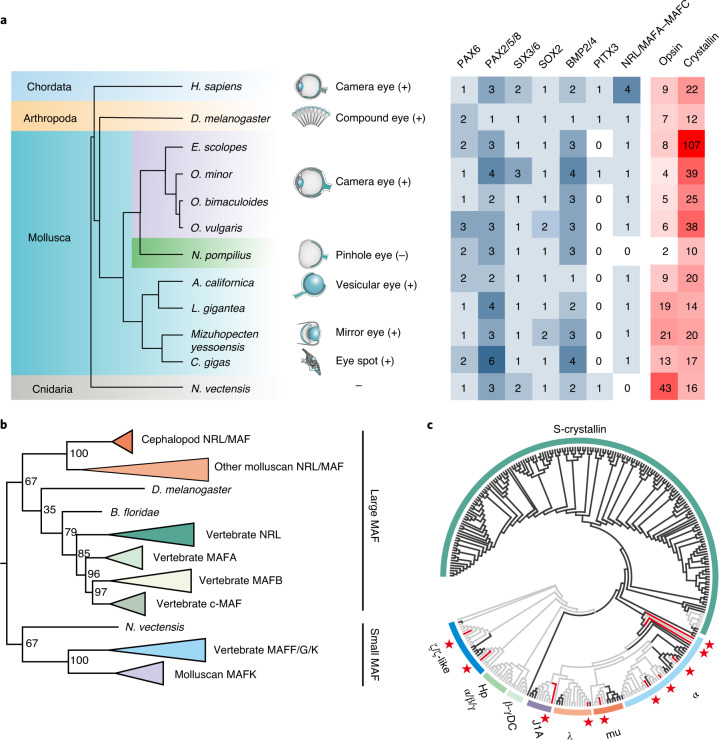
Loss of NRL and contraction of crystalline genes are linked to the evolution of the pinhole eye. **a**, Distribution of core transcription factors crucial for regulating lens development and key optic gene families in multiple metazoans; the ‘+’ and ‘−’ symbols indicate eyes with and without lenses, respectively. **b**, Phylogenetic analysis of NRL/MAF superfamily from representative metazoans. The phylogenetic tree was constructed using MrBayes under a mixed model of amino acid substitution. The degree of support for internal branching is shown as a probability percentage at the base of each node. Notably, the large MAF only preserves one copy in molluscs but diverges into four clades in vertebrates. *N. pompilius* is the only extant species that has lost NRL. **c**, Phylogenetic analysis of crystallin superfamily from representative metazoans. Coleoid cephalopods, *N. pompilius* and non-cephalopod metazoans are indicated by the black, red and grey branches, respectively. For detailed results, see Supplementary Fig. 10.

**Extended Data Fig. 4:**
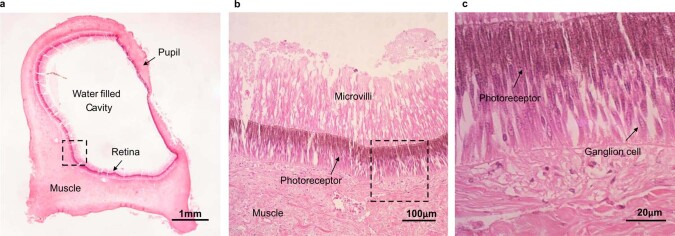
Histological analysis of the pinhole eye in *N. pompilus*. Histological features of the pinhole eye was examined in tissue sections after hematoxylin and eosin (HE) staining. Full view (panel a) and partial enlargement (panels b and c) show the photoreceptor and ganglion cells in a single optical layer.

The pinhole eye is one of the most peculiar and remarkable feature of nautilus, where an adjustable pupil instead of lens creates a relatively dim image on the retina. Vertical sections of the *N. pompilius* pinhole eye reveal that its retina contains a single layer of rhabdomeric photoreceptor cells (Extended Data Fig. 4), which is a visual sensor universally distributed in invertebrates including coleoid cephalopods^39,40^. Compared to the sophisticated camera eyes in coleoids, the relative structural simplicity of the pinhole eye highlights an excellent model for reconstructing ancient evolutionary scenarios narrating the genesis of the eye and/or lens formation. It has been postulated that changes in the ‘core regulatory complex’ of transcription factors are essential for driving the evolution of functionally specific cells or organs^41,42^. Our genomic searches for the core regulatory transcription factors governing lens formation reveal that nearly all these core regulators including PAX6, SIX3/6 and SOX2 are present in the nautilus genome (Fig. 3a). Previously, palaeontological studies reported that fossil eyes with lenses emerged during the early Cambrian, thus supporting the ancient origin of the lens^43^. Exceptionally, our comparative results indicate a lineage-specific loss of the *Nrl*/*Maf* (large *Maf*) gene in the *N. pompilius* genome (Fig. 3a and Supplementary Table 12). Phylogenetic analysis shows that molluscan *Nrl*/*Mafa*–*Mafc* belong to the large *Maf* superfamily and their orthologues diverge into four clades (*Mafa*, *Mafb*, *c-Maf* and *Nrl*) in vertebrates (Fig. 3b and Supplementary Figs. 7 and 8). Experimental evidence further supports the notion that members of the large *Maf* family are lens-specific in expression and play a central role in lens induction and differentiation in vertebrates^44,45^. Moreover, recruitment of *Nrl* or *c-Maf* can augment PAX6-induced crystallins, which are the most abundant lens structural proteins required for light refraction and transparency^46^. As expected, ten crystallin-like genes are identified in the *N. pompilius* genome and are conspicuously contracted compared to other lens-equipped molluscs (Fig. 3a). In particular, the phylogenetic tree further reveals that lineage-specific expansion of S-crystallin is found in coleoids and none of the S-crystallin genes is encoded in the *N. pompilius* genome (Fig. 3c and Supplementary Figs. 9–11), in agreement with their roles as major constitutive lens proteins in cephalopods^47^. Furthermore, investigation of transcriptional regulatory sites on crystallin proximal upstream sequences reveals that enrichment of NRL/MAF binding motif is distributed more abundantly in coleoids than in *N. pompilius* (Supplementary Fig. 12), underscoring the fact that independent gene losses in nautilus and expansion of crystallins in coleoids may be instrumental in driving eye evolution in cephalopods. However, a previous transcriptomic study reported lineage-specific loss of SIX3/6 expression in the *N. pompilius*^48^embryo, raising the possibility that alternation in core regulatory transcription factor expression may lead to evolutionary divergence of the eye.

**Fig. 4:**
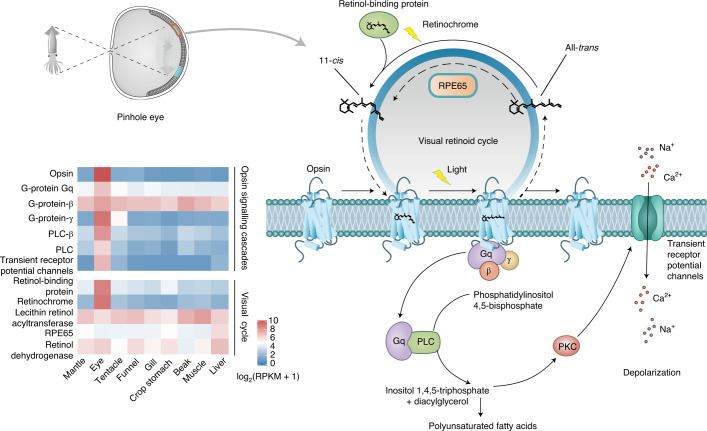
Visual model of *N. pompilius*. Key components of visual retinoid cycles and opsin signalling cascades have been identified in the *N. pompilius* genome. The heatmap of visual cycles and opsin signalling cascades indicates specific expression patterns in the eye^116,117^. PKC, protein kinase C; PLC, phosphoinositide-specific phospholipase C.

**Extended Data Fig. 5:**
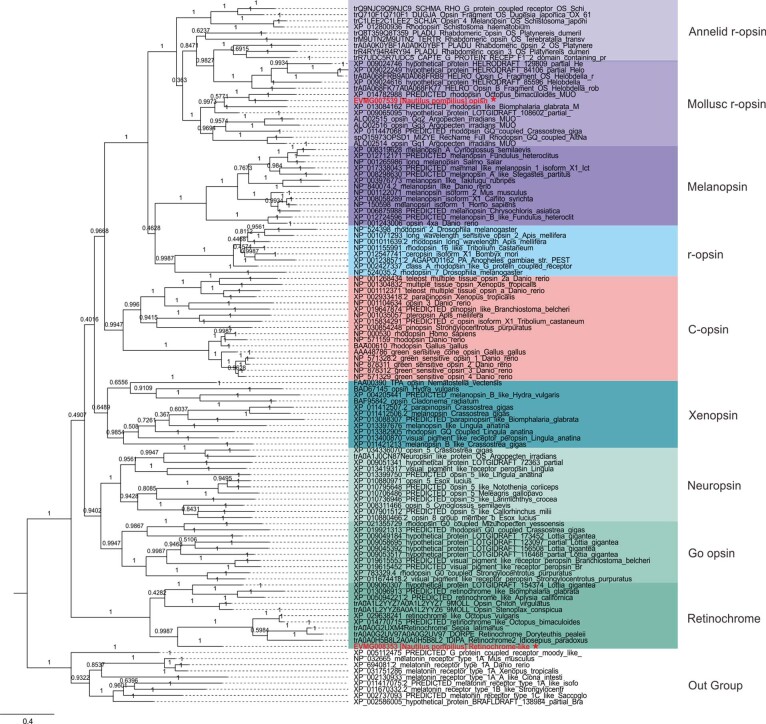
Phylogenetic tree of the opsin gene family. Phylogenetic tree was constructed by MrBayes method as described above. The melatonin receptor clade was set as an outgroup. Based on the topological structure, the ancestor of opsin divided into different clades: r-opsin (Annelid r-opsin, Mollusc r-opsin, melanopsin, and canonical r-opsin)/C-opsin/Go-opsin (Xenopsin, Nerropsin, Go opsin, and Retinochrome) clade. One r-opsin (EVMG007539) and one retinochrome (EVMG008353) were identified in the *N. pompilus* genome and marked in red.

As a nocturnal predator, nautilus has evolved the characteristic behaviour of vertical depth migration into shallower waters at night^49,50^. Understandably, light sensing and spatial vision are fundamental prerequisites for achieving this task. Phylogenetic evidence shows that the *N. pompilius* genome encodes one photoreceptive r-opsin gene and one retinochrome gene, representing the minimal opsin gene number among known metazoans (Fig. 3a and Extended Data Fig. 5). Moreover, expression pattern analysis reveals that r-opsin and its associated signalling cascades are predominantly expressed in the eye (Fig. 4), suggesting that the principal role of r-opsin lies in mediating rhabdomeric phototransduction in *N. pompilius*^51,52^. With a fair degree of certainty, monotonic r-opsin does not support colour discrimination in *N. pompilius*, suggesting colour blindness in nautilus as described in most cephalopods^53^.

**Extended Data Fig. 6:**
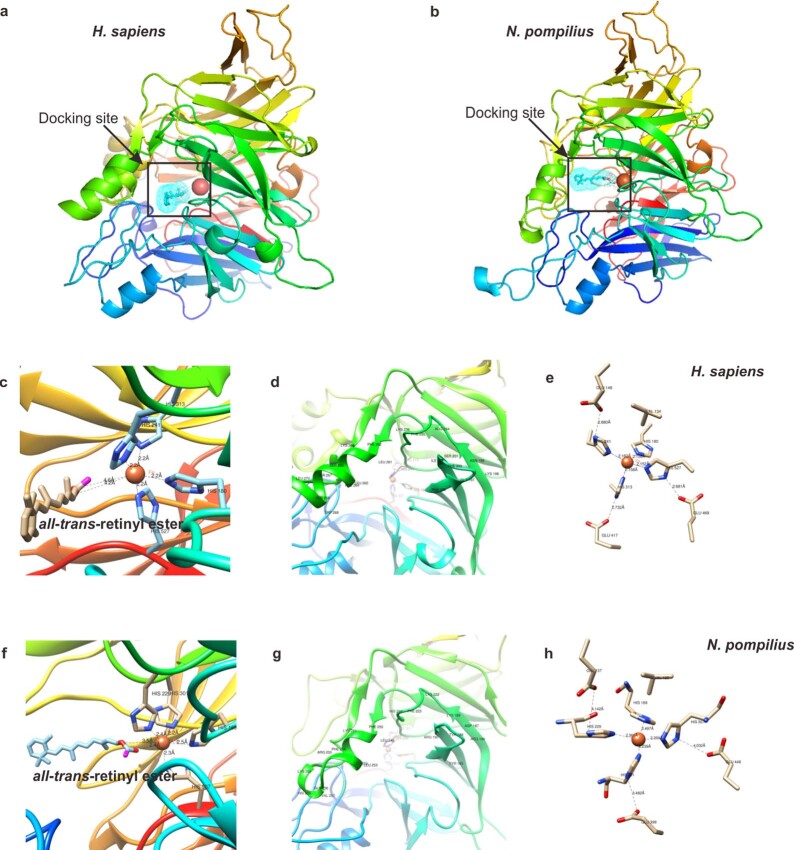
Modeling and docking of RPE65 and all-trans retinyl ester in *N. pompilius* and *H. sapiens*. Structure model of *H. sapiens* RPE65 (**a**) and *N. pompilius* RPE65 (**b**) with all-trans retinyl ester, which located near the active site defined by the iron ion. The ion cofactor is found near the top face of the propeller axis and is conserved in *H. sapiens* and *N. pompilius*, which is directly coordinated by four His residues (His^180^, His^241^, His^313^, His^527^ in *H. sapiens*; His^169^, His^229^, His^301^, His^507^ in *N. pompilius*), with average bond length of 2.16 Å in *H. sapiens*, and 2.34 Å in *N. pompilius*. Ferrous iron is required for its catalytic activity, binding to the hydroxyl oxygen to catalyze the isomerization reaction. The docking site details were displayed, revealing that a shorter average bond length (2.95 Å) between atRE and ion cofactor in *N. pompilius* (**Fig f**), than that (4.4 Å) in *H. sapiens* (**Fig c**), suggesting the catalytic potential of *N. pompilius* RPE65. The hydropholic tunnel of *N. pompilius* RPE65, leads from the protein surface to active site, the mouth of which is surrounded by three groups of residues (185–190, 222–224, and 249–259, **Fig g**), highly conserved with that in *H. sapiens* RPE65 (196–202, 234–236, and 261–271, **Fig d**). On the other hand, the *N. pompilius* RPE65 also shows a distinguishable character: the iron cofactor, ordinated by four His residues, three second shell Glu residues and a Val residue, displays a more loose structure (**Fig h**) than that in *H. sapiens* RPE65 (**Fig e**), which shows no obvious interference to its catalytic activity.

In contrast, perception of light intensity is much more critical for vertically migrating marine animals due to the dramatic decline of luminance in deep-sea waters^54^. Opsin sensitivity to light largely depends on the chromophore of 11-*cis* retinal, isomerization of which typically results in conformational changes and activation of opsin signalling transduction^55^. Thus, efficient regeneration of 11-*cis* retinal is necessary to maintain visual function^56^. In cephalopods, the retinochrome is a major and lineage-specific isomerase in the visual cycle^57^, confirmed by the identification of a retinochrome-encoded gene in the *N. pompilius* genome (Extended Data Fig. 5). Moreover, in vertebrates, retinal pigment epithelium-specific protein 65 kDa (RPE65) is a key isomerase in driving the visual retinoid cycle through converting all-*trans* retinyl ester to 11-*cis* retinol^58,59^. Intriguingly, an expansion of the *RPE65* gene family, which encodes a total of ten genes, was found and identified in the *N. pompilius* genome (Supplementary Fig. 13). In silico molecular simulation revealed that nautilus RPE65 shares a conserved iron ion-binding site, an active site cavity and a hydrophobic tunnel for substrate entry with human RPE65, thus suggesting potential catalytic activity (Supplementary Fig. 14 and Extended Data Fig. 6). Unlike restricted expression of RPE65 in pigment epithelium in vertebrates, broad expression of RPE65 across tissues including the eye was observed in *N. pompilius* in this study (Supplementary Figs. 15 and 16), which may be explained by the fact that the molluscan (including in nautilus) retina lacks an anatomical architecture similar to the pigment epithelium. From a perspective of evolutionary adaptation, the appearance of the pinhole eye is one adaptive breakthrough essential to the nautilus lifestyle of vertical depth migrations, allowing the organism to acquire spatial vision and rapidly cope with hydrostatic pressure within the eye through opening the pupil to seawater. Overall, multiple genomic innovations including gene losses, independent contraction and expansion of specific gene families and presence of associated regulatory networks seem to work in unison to drive the evolution of the pinhole eye in nautilus.

### Pearl shell formation

**Fig. 5:**
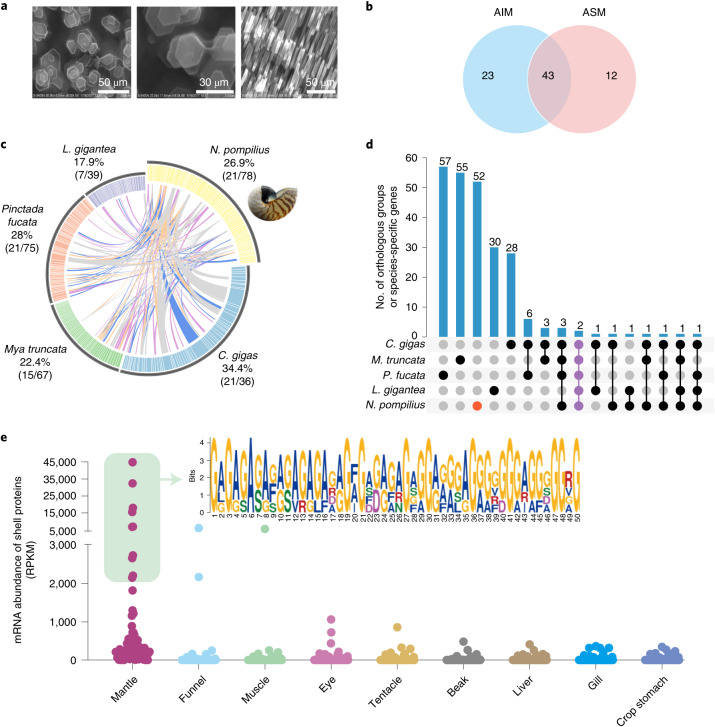
Ultrastructure and proteome of the *N. pompilius* shell. **a**, SEM images representative of the ultrastructure of the nacre layer of the *N. pompilius* shell. **b**, Number of proteins identified from the AIM and ASM fractions. **c**, Circos diagram showing similarities between five representative molluscan shell proteomes (the E*-*value cut-off of protein–protein BLAST is 1 × 10^−5^). Proteins sharing similarities between *N. pompilius* and other species are linked by different coloured lines, with the top quartile as the purple line, the second quartile as the blue line, the third quartile as the orange line and the lowest quartile as the grey line. The percentages and proportions in brackets represent the number of proteins having similarities between *N. pompilius* and four reference species. **d**, UpSet plot comparing orthologous groups and species-specific genes among five species. The red dot indicates conserved domains among the five species. **e**, Shell protein expression levels in nine tissues. The inset shows the top 10 mantle-enriched SMPs in *N. pompilius* containing new repetitive poly (Gly or Gly-Ala) motifs in de novo prediction.

**Extended Data Fig. 7:**
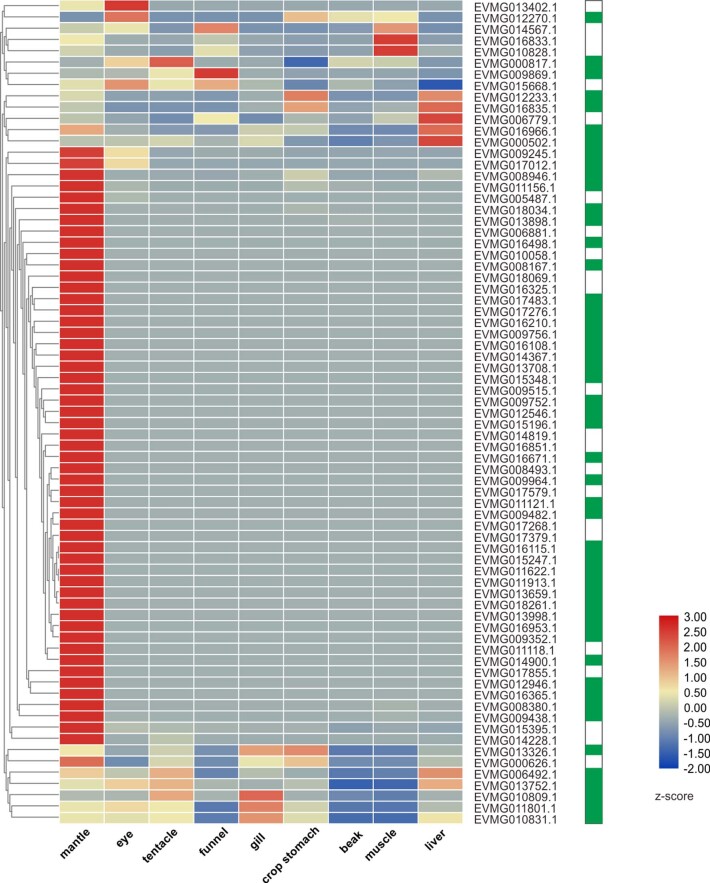
Specific expression of SMPs in the mantle of *N. pompilius*. Heatmap shows the normalized expression profiles of shell proteins in different tissues, indicating that majority of SMPs are expressed specifically and in high abundance in the mantle. Nautilus specific shell protein genes were also marked with green color in the colored bar on the right.

As the only extant cephalopod with an exoskeleton, nautilus possesses an intricate shell of spiralling chambers that not only acts as a protective physical shield against predation or environmental adversities but also plays an indispensable role in buoyancy maintenance. Thus, the unique shell architecture of nautilus results from adaptive evolution for vertical migration. Generally, molluscan shell formation is one of fundamental biomineralization processes where shell matrix proteins (SMPs) guide the growth of calcium carbonate polymorphs (calcite and/or aragonite) and organization of crystal into intricate shell formation^60^. Clearly, understanding the ultrastructural architecture and SMP biocomposition of the *N. pompilius* shell is important for uncovering the ancient mechanisms underlying shell formation and its evolution. Previous studies have assumed that the composition of aragonite crystals underpins superior strength and toughness for resisting high hydrostatic pressures in *N. pompilius*^3,61^. Our scanning electron microscopy (SEM) images of the *N. pompilius* inner layers confirm this and reveal pure aggregates of hexagonal aragonites that stack up along the direction of growth (Fig. 5a). Thus, our results lend support to the hypothesis that aragonite may be ancient crystalline calcium carbonate before calcite became the staple building blocks for the construction of the molluscan shell^62^. To further investigate the molecular basis of nautilus shell formation, a total of 78 SMPs were identified from acid-soluble (ASM) or acid-insoluble (AIM) matrix fractions derived from 2 technical replicates (Fig. 5b and Supplementary Table 13). Expression patterns showed that most of these SMPs (72.2%) were expressed especially highly in the mantle (Extended Data Fig. 7), thereby confirming a central role of the mantle in shell formation as suggested previously in molluscan species^63,64^.

**Extended Data Fig. 8:**
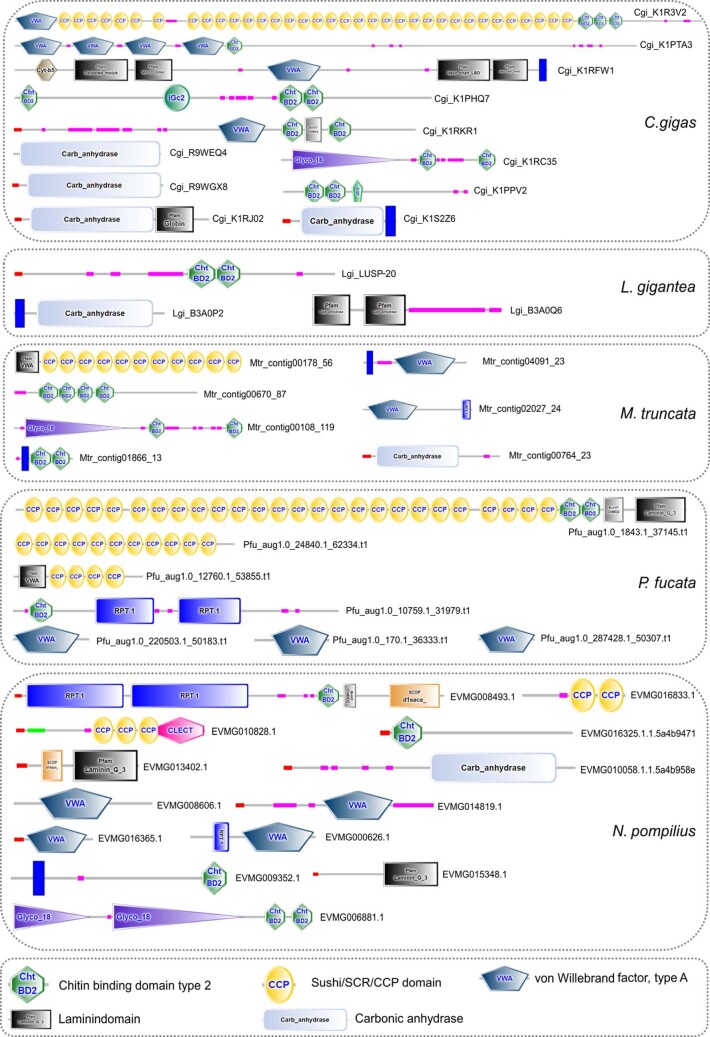
Conserved molluscan biomineralization “toolkit” among five molluscan species. The conserved domains of shell matrix proteins contain Sushi/SCR/CCP domain, laminin domain, chitin binding domain and carbonic anhydrase domain. Domain architecture was predicted and constructed by the software SMART.

To characterize the conserved molluscan biomineralization ‘toolkit’, we performed comparative shell proteomic analysis, which showed that 21 of *N. pompilius* SMPs shared similarity with counterparts in other molluscs including bivalves and gastropods (Fig. 5c). Further domain analysis revealed several conserved domains across molluscs, which contained the Sushi/SCR/CCP, laminin, chitin-binding and carbonic anhydrase domains (Extended Data Fig. 8). This evidence points to the possibility that these domains occur as an ancient ‘core biomineralization toolkit’ and are conserved across multiple molluscan lineages with an external shell^65,66^. OrthoFinder analysis showed that 52 of 78 SMPs afforded new or *N*. *pompilius-*specific shell proteins (Fig. 5d), leading us to speculate that most of the unique SMPs evolved independently and contribute to a high degree of diversity in shell architecture in molluscs. This is also supported by evidence for low similarity of the key SMP, Nautilin-63, even within the same *Nautilus* genus (Supplementary Fig. 17)^67^. Strikingly enough, we found that the top 10 mantle-enriched SMPs in *N*. *pompilius* do not match any known Pfam domains but contain new repetitive poly (Gly or Gly-Ala) motifs through de novo predictions (Fig. 5e). Therefore, the preponderance of these SMPs may be associated with the uniqueness and new features of the nautilus shell structure, further bolstering our previous assumption. Interestingly, several repetitive low-complexity domains (RLCDs) involved in aggregation or binding have been extensively identified in shell structure proteins in multiple nacre-producing bivalve and gastropod lineages^68,69^, strongly suggesting that parallel evolution of RLCDs could be a unifying principle for molluscan biomineralizaiton, especially for nacre formation.

### Immune system

**Fig. 6:**
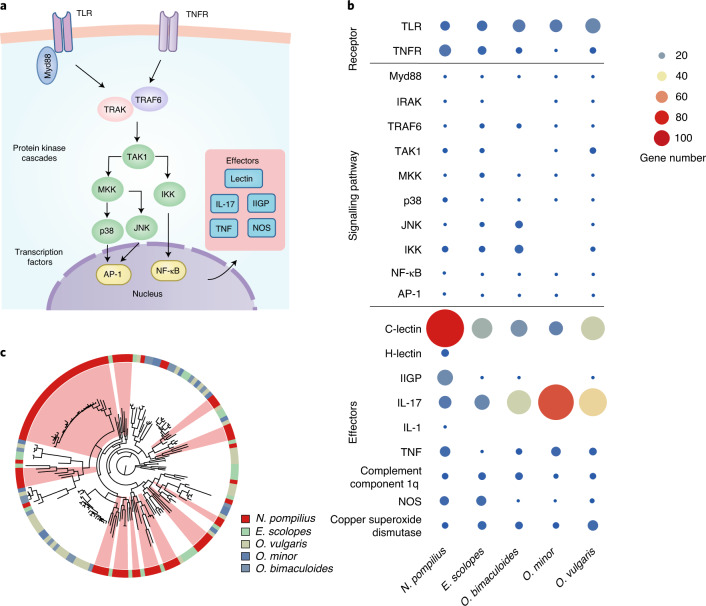
Functionally complete and specific gene expansion in the *N. pompilius* immune system. **a**, Schematic representation of molecular components in the TLR and TNFR signalling pathways. AP-1, activator protein 1; IKK, inhibitor of nuclear factor kappa-B kinase; IRAK, interleukin-1 receptor-associated kinase; JNK, c-Jun NH2-terminal kinase; MKK, mitogen-activated protein kinase kinase; Myd88, myeloid differentiation primary response 88; NF-κB, nuclear factor kappa-B; NOS, nitric oxide synthase; TAK1, transforming growth factor-β-activated kinase 1; TRAF6, TNFR-associated factor 6; TRAK, trafficking kinesin-binding protein. **b**, Distribution of TLR and TNFR signalling pathway components in representative cephalopod species. Gene numbers are represented by spheres of different sizes and colours. **c**, Phylogeny of C-type lectin in cephalopod species. The different colours in the circle represent distinct species. *N. pompilius*-specific expanded clades are labelled in light red.

To appreciate the biology of *N. pompilius*, understanding the molecular mechanisms of their immune defence is especially revealing to delineate the ancient evolutionary features of innate immunity in cephalopod ancestors. Whole-genome annotation reveals that nautilus has highly complex yet comprehensive innate immune components. In particular, Toll-like receptor (TLR) signalling and tumour necrosis factor receptor (TNFR) signalling, as the central regulators that mediate key immune responses including apoptosis, inflammation and immune defences^70,71^, are found in nautilus (Fig. 6a), suggesting an ancient origin and co-option of innate defence ‘toolkit’ genes in cephalopod ancestors. Moreover, several genes including *IL17R*, *H-lectin* and *IL1*, were specifically identified in the nautilus genome (Fig. 6b), which supports the assumption that nautilus has preserved a more complete repertoire of immune molecules than other cephalopods. Since massive duplication or expansion of key immune genes is a fundamental approach to boosting host defence^72^, we analysed the gene number of immune defence-related genes and compared distinct lineage-specific gene family expansions in nautilus and coleoids (Fig. 6b). Quite strikingly, the nautilus genome encodes a total of 81 C-type lectin genes, which is significantly expanded with regard to the 12–33 genes found in coleoids (Fig. 6b). Phylogenetic analysis further revealed that several lineage-specific lectin genes are independently duplicated in *N*. *pompilius* (Fig. 6c). In animals, lectins are versatile immune molecules indispensable for discrimination, neutralization, agglutination and destruction of pathogens via specific binding of unique carbohydrate moieties on the surface of bacteria^73^. Hence, we reason that massive expansion of lectins may have resulted in the creation of remarkable inherent diversity that is conducive to containing different pathogens emerging from dynamic environments. IFN-inducible GTPases (IIGPs), another important class of innate effectors demonstrated to play critical roles in vesicle trafficking and antimicrobial inflammasome assembly^74,75^, are also specifically expanded in the nautilus genome (Fig. 6b and Supplementary Fig. 18). Thus, an integrated, highly complex and complete innate immune system coupled to linage-specific gene expansions in nautilus contribute to the establishment of sophisticated host responses against a diverse spectrum of invading pathogens during the organism’s evolutionary history. However, we also observed that interleukin-17 (IL-17) is specifically expanded in the octopod lineage (Fig. 6b and Supplementary Fig. 19), suggesting that distinct defence mechanisms have evolved in different cephalopod linages.

## Discussion

Genomic evidence reveals that nautilus has undergone lineage-specific innovations in both body plan and behaviour since the Cambrian and retained these extraordinary features after a long evolutionary history. In particular, vertical depth migration in *Nautilus* and other chambered cephalopods is one of several critical and common strategies needed to avoid predators and budget energy; these may have helped the survival of these species ever since. The emergence of the pinhole eye is a great innovation for switching from directional to spatial vision and rapidly change hydrostatic pressure, making vertical depth migration possible. Our findings highlight that co-evolutionary loss of core regulatory transcription factors may have driven the evolution of the pinhole eye. Moreover, our proteomic and transcriptomic data suggest that an ancient ‘core biomineralization toolkit’ and new RLCDs co-ordinately directed the construction of the chamber shell, which has evolved into the buoyancy apparatus needed to adapt to a critical life mode. Taken together, the draft genome of *N. pompilius* together with multi-omics provide a valuable insight into not only the adaptive innovations of the ancestor of cephalopods but also the dynamic evolution of coleoids.

## Methods

### Sample collection and research ethics

A sample of *N. pompilius* was originally obtained via a biological resources reconnaissance survey in October 2016, during which a single adolescent individual of *N. pompilius* with a body size of 12 cm was collected near the Nansha Islands of the South China Sea (7° 62′ 7514′′ N, 112° 26′ 4571′′ E). The adolescent nautilus was then maintained in a dark tank at 16–19 °C while being transported. The organism was subsequently donated by the Chinese Ocean Conservation Association for research use in this study in accordance with local research guidelines and regulations on animal experimentation. All experimental protocols were reviewed and approved by the research ethics committee for animal experiments at the South China Sea Institute of Oceanology, Chinese Academy of Sciences. Nautilus muscle was used to extract DNA with a DNeasy Blood & Tissue Kit (QIAGEN). Multiple tissue samples including the mantle, eye, tentacle, funnel, gill, beak, muscle and liver were used for RNA extraction with the TRIzol reagent (Thermo Fisher Scientific); the quantity and quality of DNA were checked by agarose gel electrophoresis using a Qubit 2.0 fluorometer (Thermo Fisher Scientific), respectively.

### Illumina sequencing and genome size estimation

The 270-base pair (bp) paired-end libraries were constructed using Illumina’s paired-end kits according to the manufacturer’s instructions. The libraries were sequenced on an Illumina HiSeq 2500 platform. For the raw reads, sequencing adaptors were removed. Contaminated reads containing chloroplast, mitochondrial, bacterial or viral sequences were screened via alignment to the National Center for Biotechnology Information (NCBI) NR database using the Burrows–Wheeler Aligner (BWA) v.0.7.13 (ref. ^76^) with default parameters. FastUniq v.1.1 (ref. ^77^) was used to remove duplicated read pairs. Low-quality reads were filtered out on the basis of the following conditions: (1) reads with ≥10% unidentified nucleotides; (2) reads with >10 nucleotides aligned to an adaptor, allowing ≤10% mismatches; and (3) reads with >50% bases having Phred quality <5. About 59.78 gigabases (81.83×) corrected Illumina reads were selected to perform genome size estimation. *N. pompilius* genome size was estimated using the formula: genome size = *k*-mer_number/peak_depth.

### PacBio sequencing

Genomic DNA was sheared by means of a g-TUBE device (Covaris) with 20-kilobase (kb) settings. Sheared DNA was purified and concentrated with AMPure XP Beads (Agencourt) for further use in single-molecule real-time (SMRT) bell preparation according to the manufacturer’s protocol (Pacific Biosciences). The 20-kb template preparation was done by BluePippin size selection (Sage Science). Size-selected and isolated SMRT bell fractions were purified with AMPure XP Beads. Finally, these purified SMRT bells were used for primer and polymerase (P6) binding according to the manufacturer’s binding calculator (Pacific Biosciences). Single-molecule sequencing was done on a PacBio RS II platform with C4 chemistry. Only PacBio subreads equal to or longer than 500 bp were used to perform *N. pompilius* genome assembly.

### Genome assembly

#### Canu, LoRDEC and wtdbg

We used the error correction module of Canu v.1.5 (ref. ^78^) to select for longer subreads with the settings genomeSize = 753,000,000 and corOutCoverage = 109, detect raw subreads overlapping through a highly sensitive overlapped MHAP v.2.12 (corMhapSensitivity = normal) and complete error correction by the falcon_sense method (correctedErrorRate = 0.025). Then, the output subreads of Canu were further corrected using LoRDEC v.0.6 (ref. ^79^) with the parameters -k 19 -s 3 by using Illumina paired-end reads. Based on these two rounds of error-corrected subreads, we generated a draft assembly with wtdbg v.1.1.006 (https://github.com/ruanjue/wtdbg) with the parameters -t 64 -H -k 21 -S 1.02 -e 3.

#### Sparse, DBG2LOC and Canu

Trimmed Illumina 270-bp paired-end reads were assembled as contigs using the Sparse software (https://github.com/yechengxi/SparseAssembler)^80^ with default parameters. The DBG2LOC (https://github.com/yechengxi/DBG2OLC) software with the parameters KmerCovTh 2 MinOverlap 55 AdaptiveTh 0.008 k 17 RemoveChimera 1 was used to assemble the genome and combine the paired-end read assembled contigs. PacBio subreads were corrected using Canu v.1.5 as described above. The split_and_run_sparc.sh shell, created with the Sparc module and blasr software v.1.3.1 (ref. ^81^), was used to output the consensus assembly.

#### Quickmerge

The output assembly of Sparse, DBG2LOC and Canu, as a query input, was aligned against the assembly of Canu, LoRDEC and wtdbg with MUMmer v.4.0.0 (https://github.com/mummer4/mummer) with the nucmer parameters -b 500 -c 100 -l 200 -t 12 and the delta-filter parameters -I 90 -r -q and then merged using quickmerge^82^ with the parameters -hco 5.0 -c 1.5 -l 100000 -ml 5000. Finally, iterative polishing by Pilon v.1.22 (ref. ^83^) was achieved by aligning adaptor-trimmed paired-end Illumina reads to the draft assembly with the parameters --mindepth 10--changes--threads 4--fix bases.

### Evaluation of genome assembly

To evaluate genome quality, we first mapped Illumina reads onto the *N. pompilius* assembly with the BWA. Next, genome completeness was verified by mapping 248 highly conserved eukaryotic genes and 908 metazoan benchmarking universal single-copy orthologues to the genome by using BUSCO v.3.0.2b (ref. ^84^).

### Genome annotation

TE analysis was performed by building a repeat library with the prediction programs LTR_FINDER v.1.05 (ref. ^85^), MITE-Hunter v.1.0.0 (ref. ^86^), RepeatScout v.1.0.6 (ref. ^87^) and PILER-DF v.1.0 (ref. ^88^). The database was classified using PASTEClassifier v.1.0 (ref. ^18^) and combined with the Repbase database v.19.06 (ref. ^89^). TE sequences in the *N. pompilius* genome were identified and classified using RepeatMasker v.2.3 (ref. ^90^). TE divergence analysis was made by using a detailed annotation table from the output of RepeatMasker v.2.3 (ref. ^90^). By using the percentage of discrepancy between matching regions and consensus sequences in the database, we analysed the number of TEs with a certain divergence rate and built a repeat landscape using an R script that was modified from https://github.com/ValentinaBoP/TransposableElements.

Protein-coding genes were predicted based on EVM v.1.1.1 (ref. ^91^) by integrating homologue, RNA sequencing (RNA-seq) and de novo gene prediction methods. Homologue prediction was performed based on homologous peptides from *Crassostrea gigas*, *Crassostrea* *virginica*, *L. gigantea* and *Danio rerio* with GeMoMa v.1.3.1 (ref. ^92^). RNA-seq-based gene prediction was performed by mapping clean RNA-seq reads to the genome using Hisat v.2.0.4 and assembled by StringTie v.1.2.3. Multiple methods including PASA v.2.0.2, TransDecoder v.2.0 and GeneMarkS-T v.5.1 were applied to predict coding regions. GENSCAN v.20030218 (ref. ^93^), AUGUSTUS v.2.4 (ref. ^94^), GlimmerHMM v.3.0.4 (ref. ^91^), GeneID v.1.4 (ref. ^95^) and SNAP v.2006–07–28 (ref. ^96^) were used for de novo gene prediction with default parameters. UniGenes were assembled by Trinity v.Trinityrnaseq_r20131110 (ref. ^97^) and were then inputted to PASA v.2.0.2 (ref. ^98^) to predict genes. Training models used in AUGUSTUS, Glimmer HMM and SNAP were obtained from the prediction results of PASA v.2.0.2 and GeMoMa v.1.3.1. Gene models from these different approaches were combined by EVM v.1.1.1.

The predicted genes were annotated by blasting their sequences against a number of nucleotide and protein sequence databases, including COG Release 201703 (ref. ^99^), KEGG Release 20170310 (ref. ^100^), NCBI NR Release 2016_7_19 and SWISS-PROT Release 2015_01 (ref. ^101^) with an E-value cut-off of 1 × 10^−5^. Moreover, these predicted genes were annotated against the Pfam database of the HMMER v.3.1b2 software (http://www.hmmer.org) and the InterPro database of InterProScan v.5.34-73.0 (https://github.com/ebi-pf-team/interproscan). Gene Ontology for each gene was assigned by Blast2GO v.2.5 (ref. ^102^) based on NCBI databases.

### Phylogenetic analysis, gene expansion and contraction

Protein sequences of *Branchiostoma floridae* (GCF_000003815.1), *L. gigantea* (GCF_000327385.1), *A. californica* (GCF_000002075.1), *Tribolium castaneum* (GCF_000002335.3), *C. gigas* (GCF_000297895.1), *Helobdella robusta* (GCF_000326865.1), *Capitella teleta* (GCA_000328365.1), *Chlamys farreri* (CfBase), *Nematostella vectensi*s (GCF_000209225.1)*, E. scolopes* (GCA_004765925.1), *O. bimaculoides* (GCF_001194135.1), *Octopus minor* (GigaDB), *O. vulgaris* (CephRes-gdatabase), *Drosophila melanogaster* (FlyBase), *Homo sapiens* (hg38) and *N. pompilius* comprising 388,531 protein sequences were clustered into 40,231 orthologue groups using OrthoMCL v.3.1 (ref. ^103^) based on an all-versus-all BLASTP strategy with an E-value of 1 × 10^−5^ and a Markov chain clustering default inflation parameter of 1.5. To construct phylogenetic relationships, 423 single-copy orthologues were extracted from all 16 species and multiple alignment analysis was performed with MUSCLE v.3.8.31 (ref. ^104^). All alignments were combined into one supergene and a phylogenetic tree was analysed with RAxML v.8.2.12 (ref. ^105^) with 1,000 rapid bootstrap analyses, followed by searching for a best-scoring maximum likelihood tree in 1 single run. Finally, divergence time was estimated using MCMCTree from the PAML package v.4.7a (ref. ^106^) in combination with a molecular clock model. Several reference-calibrated time points referring to the TimeTree database (http://timetree.org/) (Supplementary Table 14). Homologue clusters with >100 gene copies in 1 or more species were separated from the OrthoMCL results. Expansion and contraction of the reserved homologue clusters were determined by CAFE v.4.2 (ref. ^107^) calculations with the parameters lambda -s and *P* < 0.01 on the basis of changes in gene family size with regard to phylogeny and species divergence time.

### Evolutionary rate test

To compare the relative evolutionary rates of *N. pompilius* with other cephalopods, 1,223 one-to-one orthologues between 5 cephalopods species were identified with the InParanoid v.4.1 software (http://inparanoid.sbc.su.se) from 5 cephalopod species and *L. gigantea*. Then, these 1,223 orthologous proteins were aligned with MUSCLE v.3.8.31 and concatenated into a super alignment. Among them, *L. gigantea* was assigned as an out-group. Tajima’s relative rate test analysis was conducted using MEGA v.7.0.18 (ref. ^108^).

To compare the neutral nucleotide mutation rate for *N. pompilius* relative to other cephalopods, alignment of the 4D sites of 1,223 one-to-one orthologues from 5 cephalopods and 1 out-group (*L. gigantea*) was performed. The results were used in the topology obtained from our phylogenetic analysis as an input for RAxML v.8.2.12 (ref. ^105^) optimization of branch lengths in 4D alignment. Pairwise distances to *L. gigantea* were calculated from the neutral tree by using the cophenetic function implemented in the R package ape v.3.2.

### Exon and intron evolution in cephalopod species

The 1,223 orthologous proteins of 5 cephalopod species were aligned using MUSCLE v.3.8.31. The position of introns longer than 50 nucleotides and characteristic of U2 or U12 splicing boundaries were mapped out using a customized Perl script. In addition, 3,071 discordant intron positions were identified based on previous methods^109^, the distributions of which were determined based on their phylogenetic relationship. Intron gains and losses were inferred by phylogenetic distributions using parsimony.

### Population size estimation

The demographic history of *N. pompilius* was analysed with the PSMC v.0.6.5 software^110^. The synonymous mutation rate per base per year was inferred based on the formula *T* = *ks*/(2*λ*). The generation time was assumed to be 15 years in *N. pompilius* and 3 months to 1 year in other cephalopods (Supplementary Table 15).

### *Hox* gene analysis

The structure of *Hox* genes in the *N. pompilius* genome was analysed with GeMoMa v.1.4.2 (ref. ^111^) using default parameters and based on available *Hox* gene models. Predictions were made by applying a GeMoMa annotation filter with default parameters, with the exception of the evidence percentage filter (e = 0.1). These were then manually verified to achieve a single high-confidence transcript prediction per locus. The exact annotations of each *Hox* gene were completed using phylogenetic relationships.

### Analysis of eye development genes

Key transcription factors and genes for eye development in the human genome were used as queries to identify their orthologues in other lineages. For lineage-specific gene families, such as S-crystallin, queries were set as homologues in the genome of *O. bimaculoides*. First, homologous searches in the gene set were performed using BLASTP with an E-value of 1 × 10^−5^. Then, the identified candidates were aligned back to the human gene set; only orthologues with the best BLASTP hit matches were defined as orthologues in each species. Additionally, TBLASTN was used to avoid any omissions in genome annotation. The accession numbers of these protein sequences are listed in Supplementary Table 12.

### Transcriptomic analysis

Total RNA was isolated from different tissues of *N. pompilius* and treated with RNase-free DNase I (Promega Corporation), according to the manufacturer’s protocol. The quality and integrity of RNA were checked using an Agilent 2100 Bioanalyzer. Illumina RNA-seq libraries were prepared and sequenced on a HiSeq 2500 system with a PE150 strategy, according to the manufacturer’s instructions (Illumina). After trimming based on quality scores using Btrim v.0.2.0, clean reads were aligned to the *N. pompilius* genome with TopHat v.2.1.1 (ref. ^112^). Gene abundance in different tissues was calculated using Cufflinks v.2.1.1 (ref. ^113^).

### SEM

To characterize crystal structures, precleaned *N. pompilius* shells were fractured and carefully collected with a dissecting knife. Pieces of fractured ligaments were dried with liquid nitrogen at a critical point followed by platinum coating using a sputter coater. Then, the shell surface was examined by SEM (S-3400N; Hitachi) with an accelerating voltage of 30 kV in high vacuum mode.

### Isolation of shell proteomics

SMPs were extracted from *N. pompilius* shells according to a protocol described previously with minor modifications^114^. First, shells were processed using abrasive paper to remove organic contaminants on the surface and washed with Milli-Q three times. Then, shells were immersed in 5% NaClO for 24 h under 4 °C with gentle shaking, washed three times with Milli-Q and air-dried at room temperature. Shells were ground into a powder and sieved by means of a nylon mesh (200 μm). Afterwards, the shell powder was bleached using 10% NaClO for 5 h. The mixture was then centrifuged at 3,000 r.p.m. for 10 min at 4 °C to remove the supernatant, washed twice and freeze-dried. The precleaned shell powder was titrated using 10% acetic acid at 4 °C with gentle shaking until all calcified constituents were completely dissolved. The powder solution was centrifuged again at 1,000 r.p.m. for 10 min at 4 °C to yield supernatant (an ASM) and precipitate (an AIM) fractions. The AIM fraction was further washed twice in Milli-Q, lyophilized and reconstituted with 8 M of urea (with 2% SDS). Both AIM and ASM were concentrated using an Amicon Ultra 3 K centrifugal filter, purified with methanol/chloroform and further reconstituted in 8 M of urea.

Since the concentrations of AIM and ASM proteins were quite low, we adopted an in-solution digestion method. Briefly, proteins were reduced by dithiothreitol with a final concentration of 10 mM at 56 °C for 1 h. The exposed sulphhydryl groups were then alkylated by 55 mM of iodoacetamide for 30 min at room temperature. After being diluted eightfold with 50 mM of triethylammonium bicarbonate, the sample solutions were digested for 16 h at 37 °C using sequencing-grade trypsin (Promega Corporation), desalted via Sep-Pak C18 cartridges (Waters Corporation) and dried off in a vacuum concentrator. The dried samples were then reconstituted in 0.1% formic acid for analysis by a LTQ Orbitrap Elite system coupled to an EASY-nLC (Thermo Fisher Scientific), as described elsewhere^115^. The .mgf files converted from raw liquid chromatography–tandem mass spectrometry data files using Proteome Discovery 1.3.0.339 (Thermo Fisher Scientific) were searched against Mascot v.2.3.2 (Matrix Sciences). The database included both target and decoy sequences of the *N. pompilius* protein database. Proteins detected in two replicates were kept for further analysis.

### Reporting Summary

Further information on research design is available in the Nature Research Reporting Summary linked to this article.

## Supplementary information

Supplementary Information Supplementary Figs. 1–19.

Reporting Summary

Supplementary Tables Supplementary Tables 1–15.

## Data Availability

The nautilus genome project has been deposited with the NCBI under the BioProject number PRJNA614552. The whole-genome sequencing data were deposited with the sequence read archive (SRA) database under accession nos. SRR11485669–SRR11485706. The RNA-seq data from various tissue transcriptomes have also been deposited with the SRA database under accession nos. SRR11485678–SRR11485687. Gene annotation data have been deposited in the Genome Warehouse database of the Genome Sequence Archive (GSA) under accession no. GWHBECW00000000.
