# Supplementary material for: The genome of Nautilus pompilius illuminates eye evolution and biomineralization
Source: Nat Ecol Evol. 2021 May 10;5(7):927–38. doi: 10.1038/s41559-021-01448-6 (PMC8257504; doi:10.1038/s41559-021-01448-6)
Supplement: Supplementary file 1 — Supplementary Figs. 1–19. [file 41559_2021_1448_MOESM1_ESM.pdf]

nature portfolio

https://doi.org/10.1038/s41559-021-01448-6

Supplementary information

The genome of *Nautilus pompilius* illuminates eye evolution and biomineralization

In the format provided by the authors and unedited

Supplementary Figures

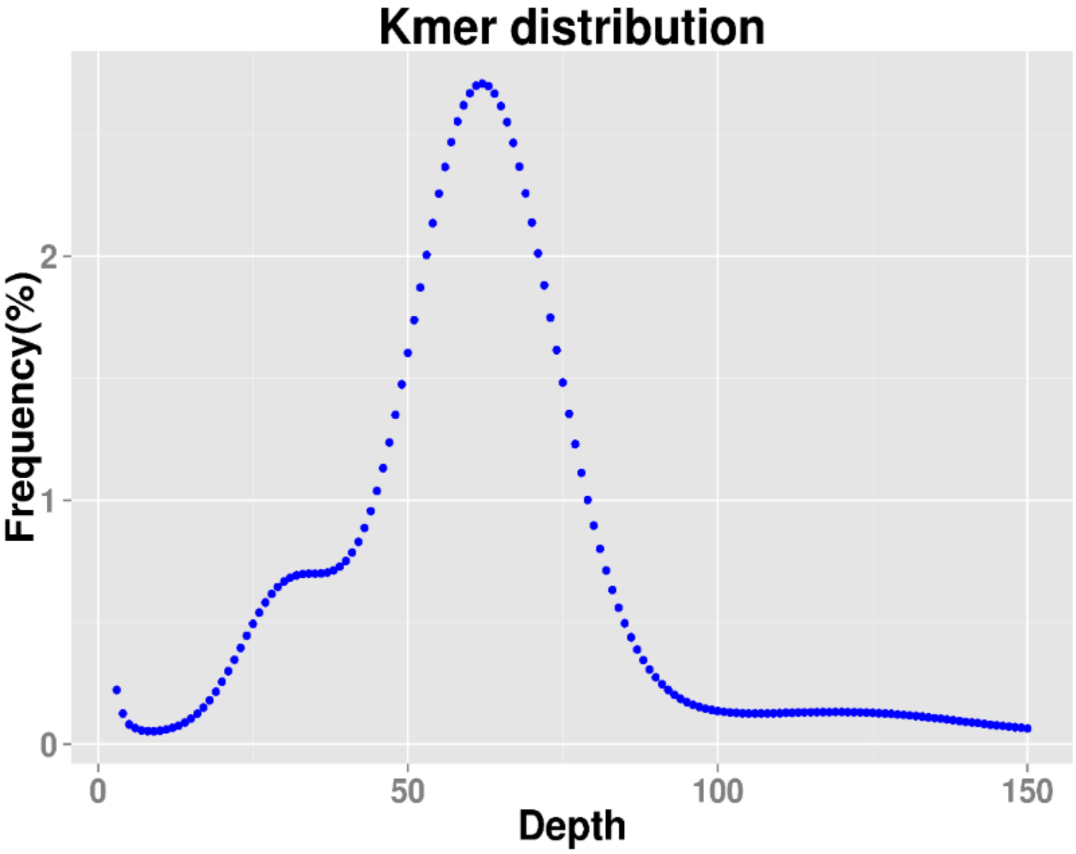

Supplementary Fig. 1 | k-mer distribution of the *N. pompilius* genome. Genome size estimation was performed by the k-mer analysis, and about 59.78 Gb corrected Illumina reads were selected to estimate the genome size. The genome size of *N. pompilius* thus estimated is 753.09 Mb.

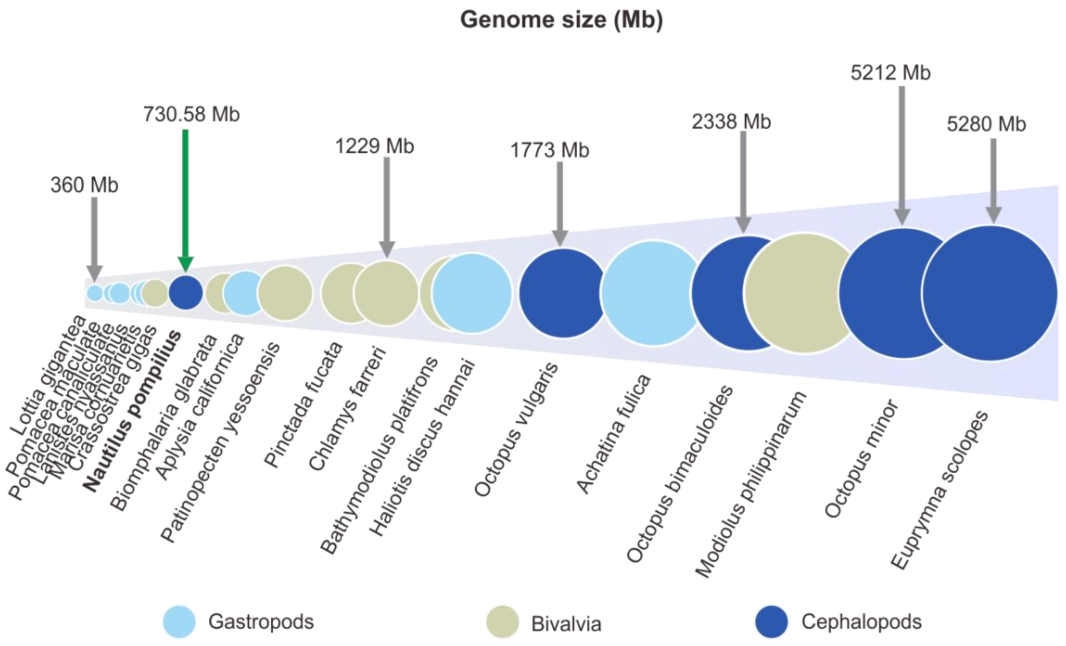

Supplementary Fig. 2 | Distribution of genome size in different molluscan lineages. Molluscan species are lined up according to their genome sizes, ranging from 360 Mb (*L. gigantea*) to 5.28 Gb (*E. scolopes*)[1-11]. Gastropods, bivalvia and cephalopods are indicated by different colors. Notably, the genome size of *N. pompilius* is the smallest among known cephalopods.

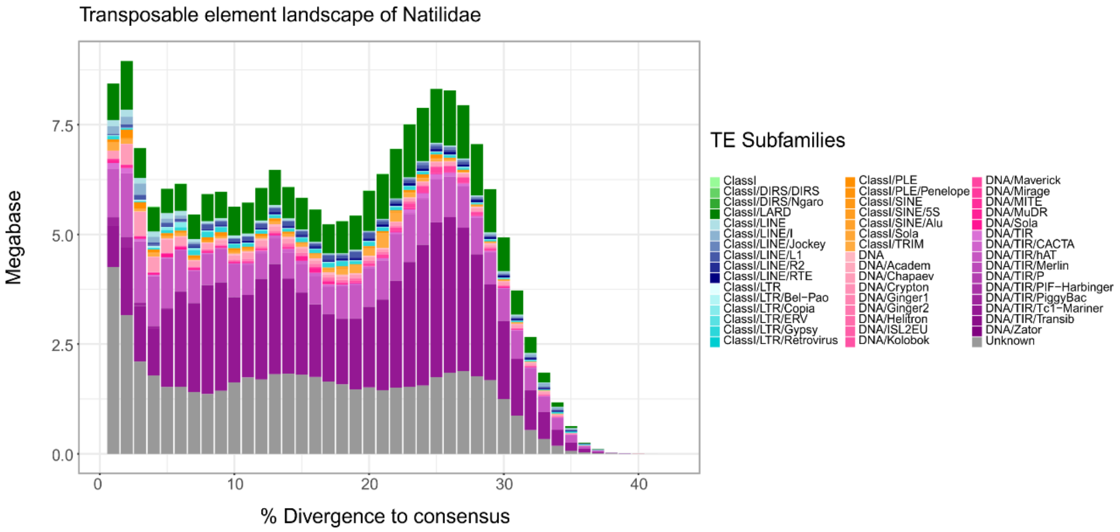

Supplementary Fig. 3 | History of transposable element (TE) accumulation in the *N. pompilius* genome. Temporal changes in transposable element (TE) accumulation in the *N. pompilius* genome based on a Kimura distance-based copy divergence analysis of TEs, with Kimura substitution level (CpG adjusted) illustrated on the *x*-axis, and percentage of the genome represented by each repeat type on the *y*-axis. Repeat type is indicated by different colored bars.

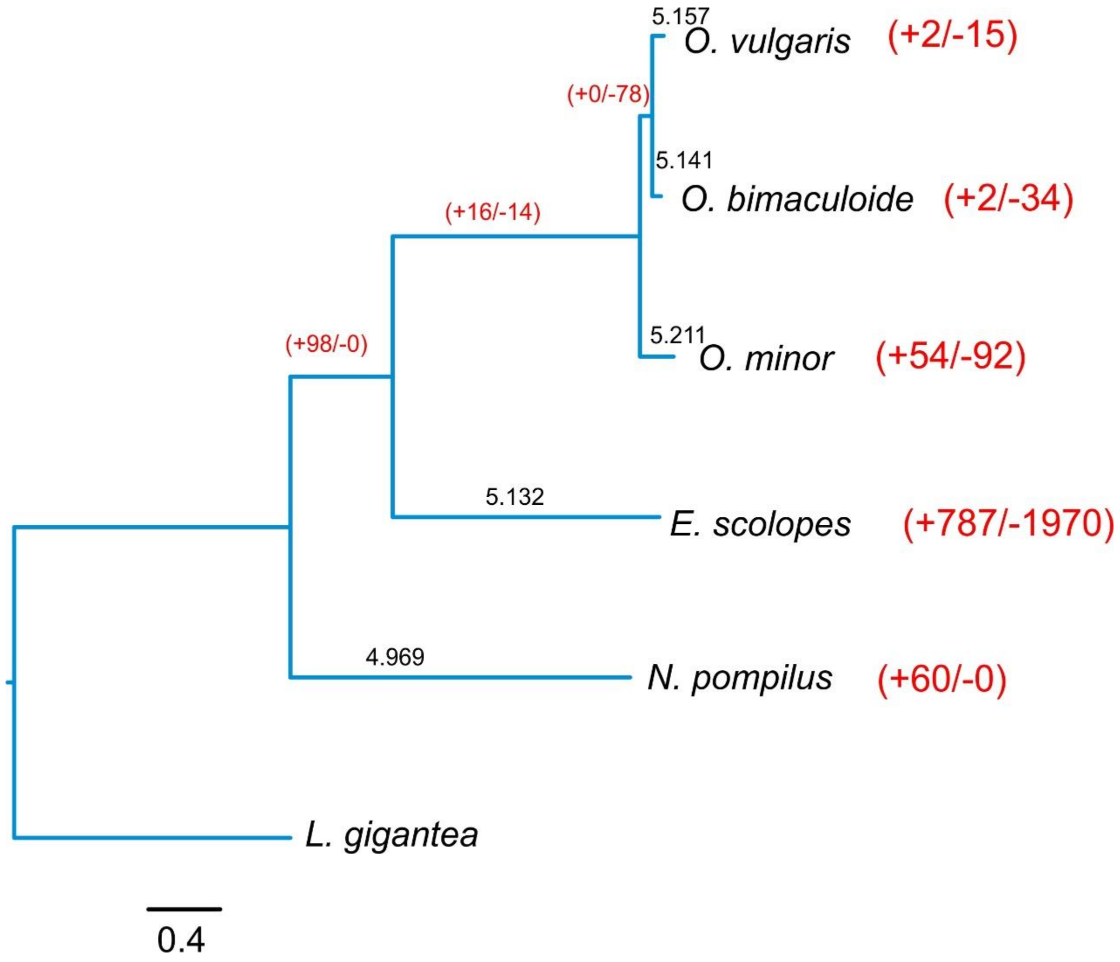

Supplementary Fig. 4 | Neutral tree and intron gain/loss event. Neutral tree of five cephalopods and *L. gigantea* is based on fourfold degenerate sites and pairwise distances to *L. gigantean* are shown for each species above their respective branches. Intron gain/loss events are shown in red besides taxon labels and at the ancestral nodes.

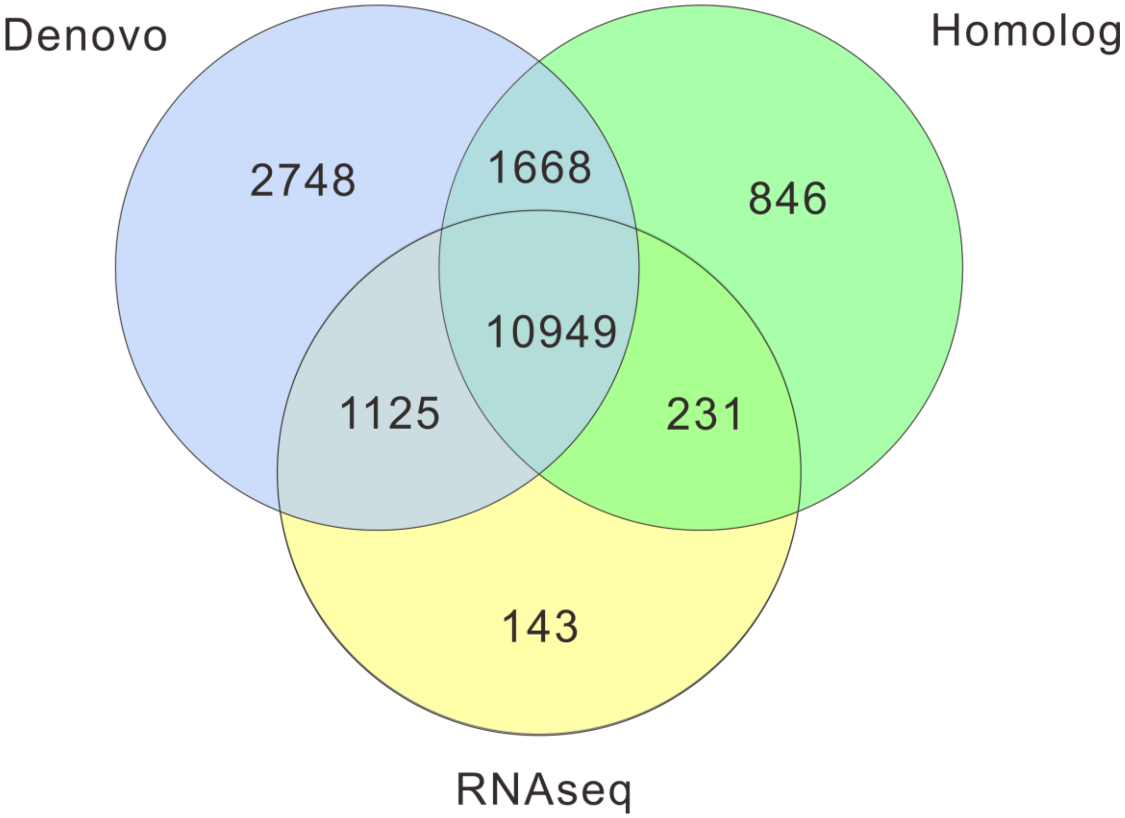

Supplementary Fig. 5 | Genome annotation in *N. pompilius.* Whole-genome annotation was performed by integrating multiple methods, which eventually generated 17,710 protein coding genes.

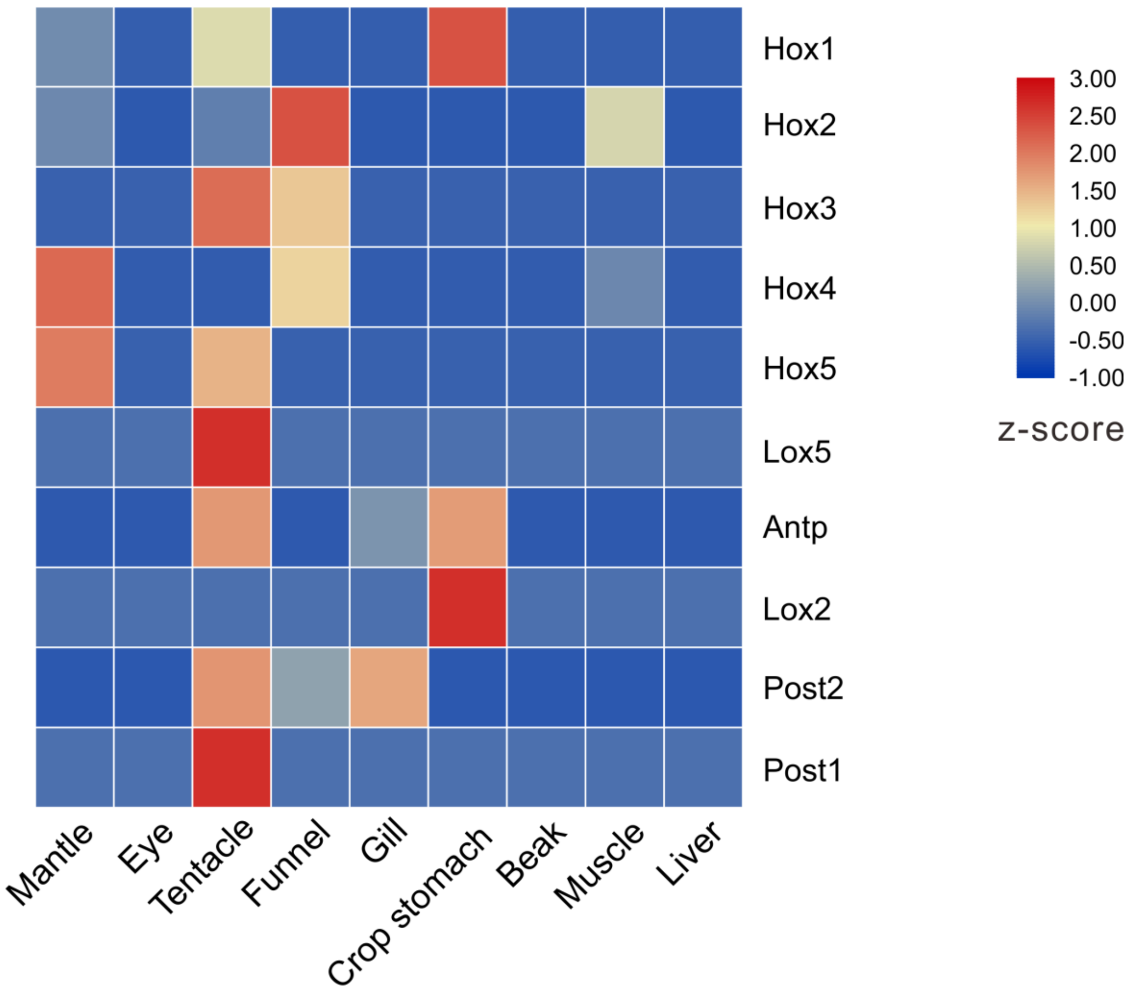

Supplementary Fig. 6 | Tissue distribution of Hox cluster in *N. pompilius*. Heatmap shows the expression profile of Hox cluster genes in different tissues. *x*-axis displays different tissues and *y*-axis shows the degree of expression of different Hox genes. Colored bars represent *Z*-score calculated from RPKM-values of a target gene in different tissues.

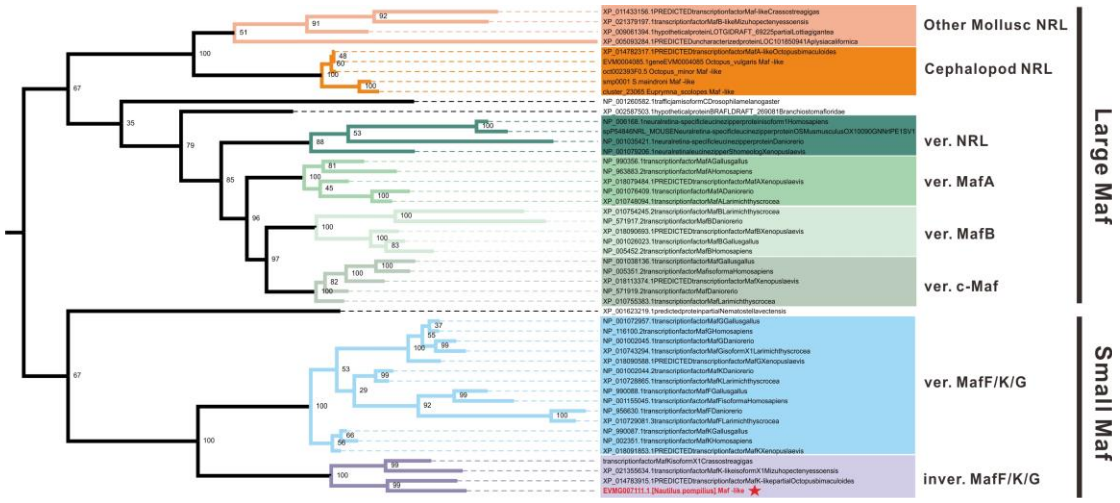

Supplementary Fig. 7 | Phylogenetic tree of the Maf/NRL superfamily. Multiple alignment was performed by using three methods including MAFFT 7.221[12], MUSCLE[13] and T-coffee[14]. The best alignment was applied to phylogenetic analysis beads on MUMSA scores[15]. Then, the phylogenetic tree was constructed by MrBayes 3.2.1[16] under a mixed model of amino acid substitution. Two independent runs with one cold and three heated chains were set for 15,000,000 generations. Starting trees were random and the trees were sampled every 1,000th generation. The ancestor of Maf/NRL was divided into the large and the small Maf clades. Each clade evolves independently and expands specifically in vertebrates. In the large Maf clades, the ancestor of Maf was continuously duplicated three times and generated four members (NRL, Maf A, Maf B and c-Maf) in vertebrates, but preserved one copy of NRL in mulloscan lineages. Similarly, the small Maf clade was divided into Maf F, Maf G and Maf K after vertebrate-specific duplications, while one copy of Maf K was preserved in mulloscan lineages. In contrast, the *N. pompilius* genome only encodes one Maf K gene but lost NRL. ver., vertebrate; inver., invertebrate.

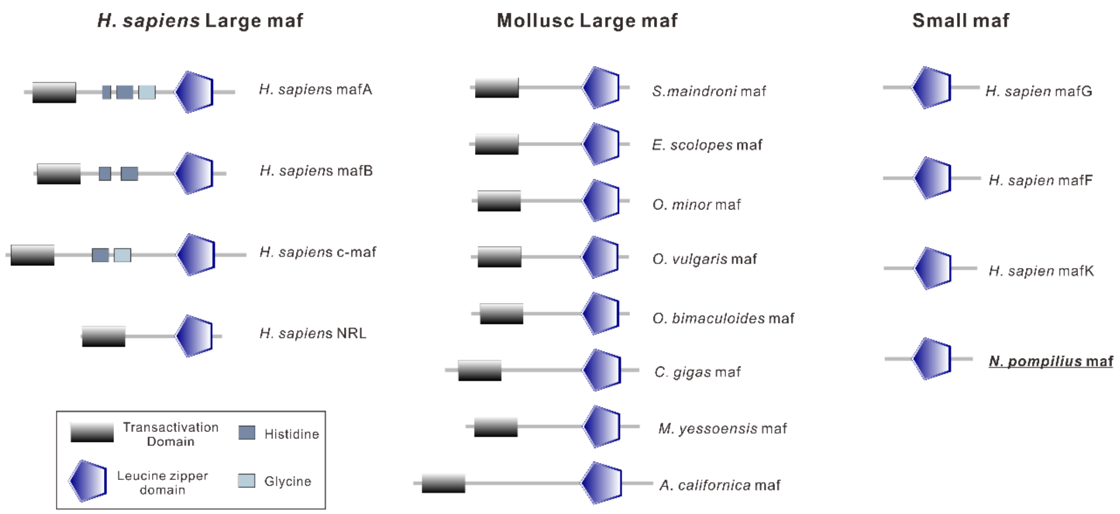

Supplementary Fig. 8 | Domain composition of Maf/NRL family members across different metazoans. MafA, MafB, c-Maf and NRL belong to large Maf family, and Maf K Maf G and Maf F belong to the small Maf family. The only extant homologue of Maf in the *N. pompilius* genome is the member of small Maf. Domain architecture was predicted and constructed by the software SMART[17].

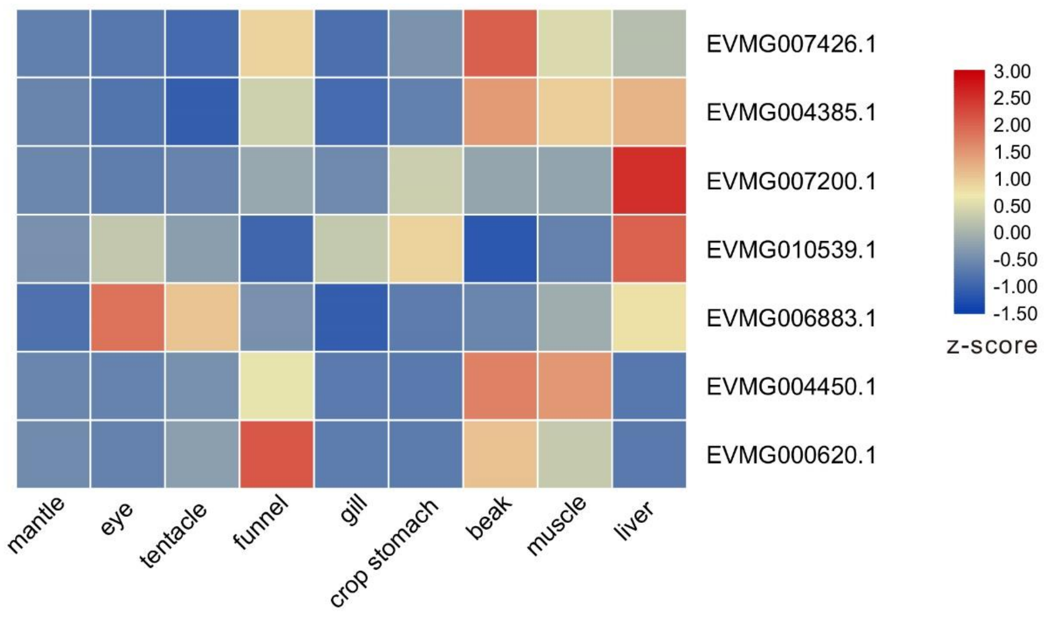

Supplementary Fig. 9 | Tissue distribution of crystallin-like genes in *N. pompilius*. Heatmap shows the expression profile of crystallin genes in different tissues, in which 3 of crystallin genes without expression are excluded. *x*-axis displays different tissues and *y*-axis shows the degree of expression of different crystallin genes. Colored bars represent *Z*-score calculated from RPKM-values of a target gene in different tissues.

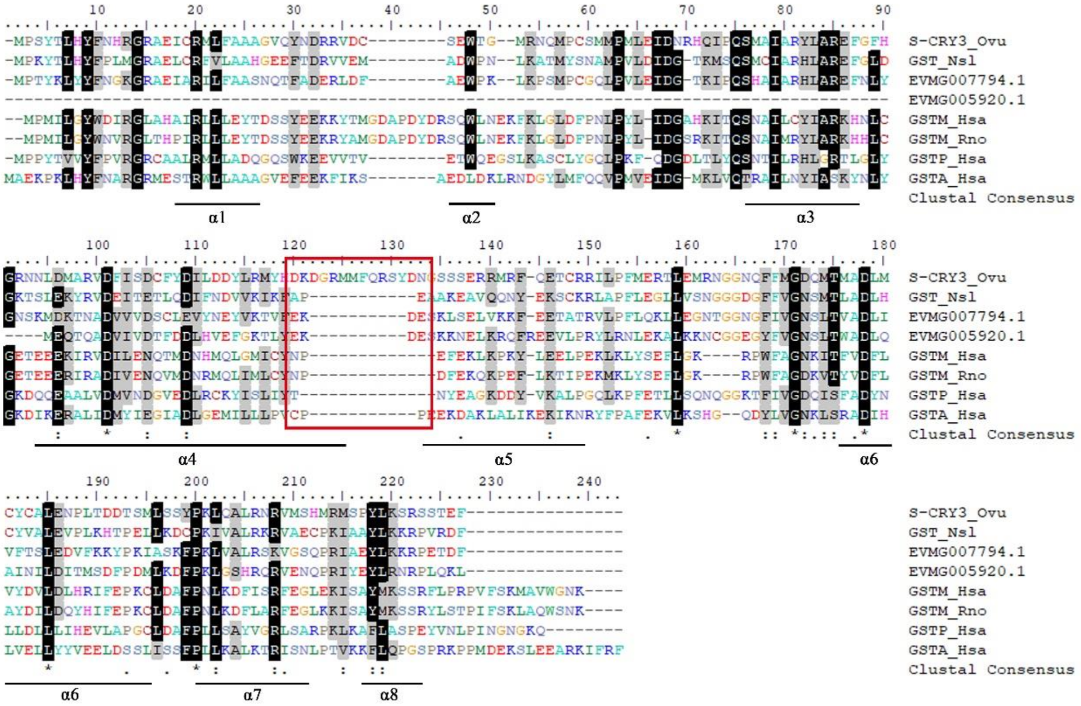

Supplementary Fig. 10 | Pairwise alignment of the potential S-crystallin of *N. pompilius* with cephalopods S-crystallin, Glutathione S-transferase (GST) and other classes of GST. The a-helices in S-crystallin are underlined and labeled. Compared with other classes of GST, the cephalopods S-crystallin has an 11-amino acid residues insertion between the conserved a4 and a5 helices (red box). Ovu, *Octopus vulgaris*; Nsl, *Nototodarus sloanii*; Has, *Homo sapiens*; Rno, *Rattus norvegicus*.

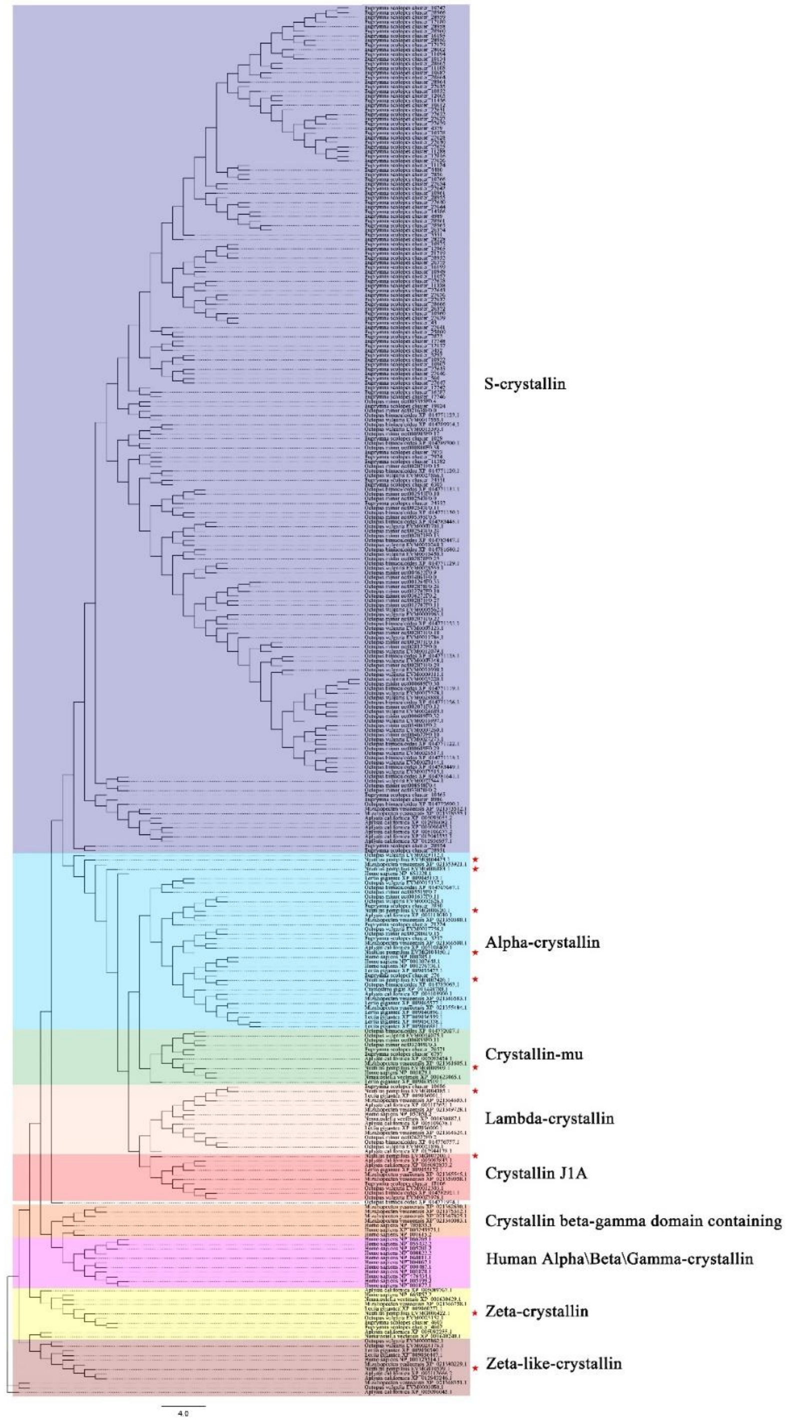

Supplementary Fig. 11 | Phylogenetic tree of the crystallin gene family. Phylogenetic tree of crystallin family were constructed by MrBayes methods as described above. Crystallin genes from *Homo sapiens*, *Euprymna scolopes*, *Octopus minor*, *Octopus bimaculoides*, *Octopus vulgaris*, *Nautilus pompilius*, *Aplysia californica*, *Lottia gigantea*, *Mizuhopecten yessoensis*, *Crassostrea gigas* and *Nematostella vectensis* are used. Different types of crystallin are labeled with separate colors. *N. pompilius* genome only contains a total of 10 crystallin genes (by red pentacle) and lacking S-crystallin which constitutes the major lens protein in cephalopods, featuring the least number of crystallins in metazoans.

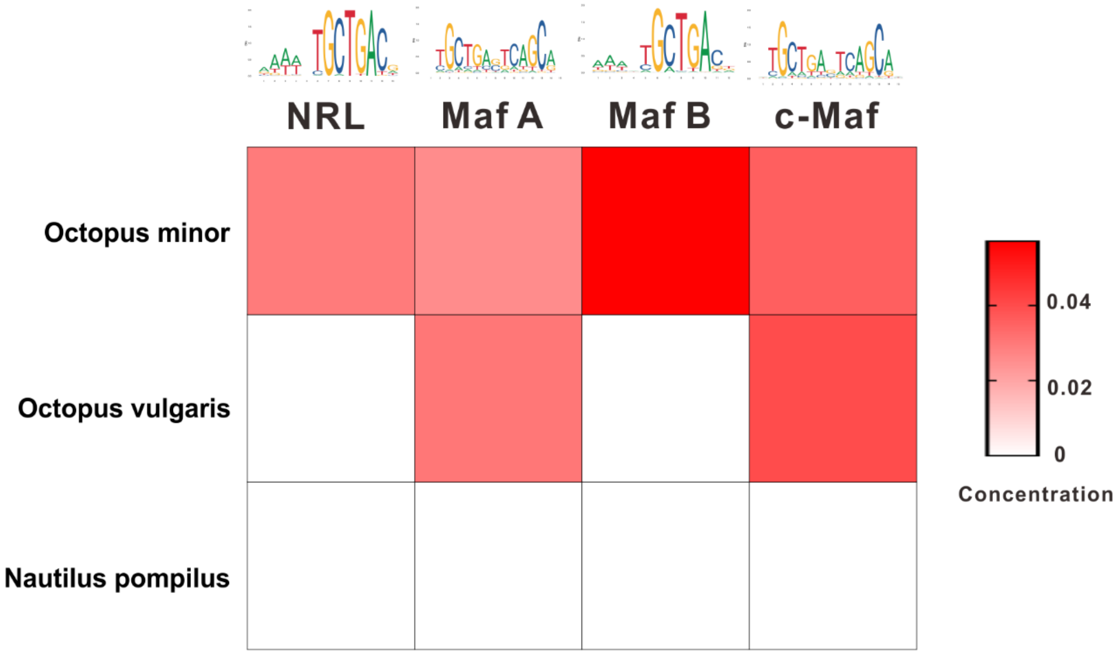

Supplementary Fig. 12 | Enrichment analysis on NRL/MAF binding motifs on the promoter of the cephalopod crystallin gene family. 2,000 bp of 5'-flanking regions of crystallin genes were extracted from the genomes of *O. minor, O. vulgaris* and *N. pompilus*. NRL, Maf A, Maf B and c-Maf binding motif matrices were downloaded from a JASPAR database. Enrichment analysis for NRL/MAF bingding motifs in the crystallin promoter regions were analyzed by CentriMo[18]. Search parameters are set as follows: (1) 0-order background model generated from supplied sequences; (2) motif sites on either strand is considered; (3) motif sites only are considered, if they have a match score ≥ 5; and (4) regions are only reported, if they have a *E*-value ≤ 1.

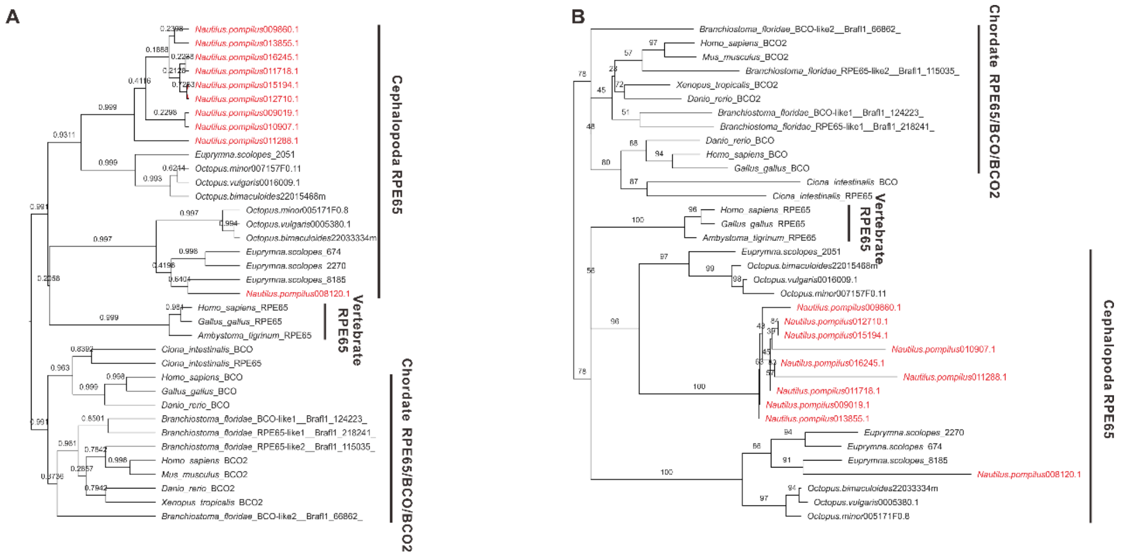

Supplementary Fig. 13 | RPE65 family expansion in *N. pompilus*. RPE65 domain containing proteins in cephalopods were applied to construct a phylogenetic tree by MrBayes method (A) as described above and ML method (B, model: LG+G4; bootstrap: 1000), respectively. The cephalopod RPE65 are homologs of vertebrates RPE65. Moreover, the *N. pompilus* genome contains a total of 10 RPE65 proteins, among which 9 of them were expanded and specifically clustered into one independent clade, and 1 of them was clustered with coleoids and formed one other clade.

```
                            10         20         30         40         50         60         70
                    ....|....| ....|....| ....|....| ....|....| ....|....| ....|....| ....|....|
H. sapiens          ---------- -MSIQVEHPA GGYKKLFETV EELSSPLTAH VTGRIPLWLT GSLLRCGPGL FEVGSEPFYH  59
EVMG013855.1    1   ---------- ---------- MALVRSFSLP LETKQPVKTT ITGSIPHWLS GSLFRNGPGV QKVDGFKLNH  50
EVMG012710.1    1   ---------- ---------- ---------- ---------- ---------- ---------- ----------  1
EVMG008120.1    1   MKPLLILTVF IIVAKAEDPD VGFNLLYTSN EKEFRDVPVR FEYPLPKWLE GTLVRNGGGG FEMGKRKLIH  70
EVMG016245.1    1   ---------- ---------- ---------- ---------- ---------- ---------- ----------  1
EVMG009860.1    1   ---------- ---------- MALVRSFSLQ LETKRPVKTT ITGSIPHWLS GSLFRNGPGV QKVDGFKLNH  50
EVMG010907.1    1   ---------- ---------- MSSHSDGCFP DQEQHPAQP- --------FT LDVPTADIGS QKVDGFKLNH  41

                            80         90        100        110        120        130        140
                    ....|....| ....|....| ....|....| ....|....| ....|....| ....|....| ....|....|
H. sapiens      60  LFDGQALLHK FDFK-EGHVT YHRRFIRTDA YVRAMTEKRI VITEFGTCAF PDPCKNIFSR FFSYFRGVEV  128
EVMG013855.1    51  LFDGFAVVHR FDIK-DREVL YQNKILKTED WLFAIKTHRL LATHFGTVPV PDPCKCLFSR HFSFYF--KM  117
EVMG012710.1    1   -------MYQ NKIL-KTEDW LS-------- ---AA HRLL ATQLV----- -------LYL HFSFYFNSKR  38
EVMG008120.1    71  AFDAYSKLTS WKFHGNGSVS FSTVFLKTES YNRSAASQDV APYLLLLGVN PP-----FSA LQRTAALLRG  135
EVMG016245.1    1   ---------- ---------- ---------- ---------- ---------- ---------- ----------  1
EVMG009860.1    51  LFDGFAVVHR FDIK-DGEVM YQNKILKTED WLSAIKTHRL LATQFGTVSV PDPCKCLFSR HFSFYFNSKR  119
EVMG010907.1    42  LFDGFAVVHR FDIK-DGEVI LV-------- ----VCCSPL VSDSVGTVSV PDPCKCLFSR HFSFYFNSKR  98

                           150        160        170        180        190        200        210
                    ....|....| ....|....| ....|....| ....|....| ....|....| ....|....| ....|....|
H. sapiens      129 TDNALVNVYP V-----GEDY YACTETNFIT KINPETLETI KQVDLCNYVS VNGATAHPHI ENDGTVYNIG  193
EVMG013855.1    118 TDNVSVNIFK H-----GDGL FAVSEIDNIW RIDPQSLGTV EKTAVSDHMA VHMATAHPLI DRNGMIYNVG  182
EVMG012710.1    39  TDNVSVNIFR H-----GDGL FAVSEIDNIW RIDPQSLDTV EKTAVSDHMA VHMATAHPLI DRNGMVYNVG  103
EVMG008120.1    136 IDNMNVNVFR YPTDDGGTGY FALNDYWKVY EFSIGRLDVL GGPVNPPIFS RPPASPGAEI GVLSQISSAH  205
EVMG016245.1    1   ---------- ---------- ---------- ---------- --------MA VHMATAHPLI DRNGMVYNVG  22
EVMG009860.1    120 TDNVSVNIFR H-----GDGL FAVSEIDNIW RIDPQSLDTV EKTAVSDHMA VHMATAHPLI DRNGMVYNVG  184
EVMG010907.1    99  TDNVSVNIFR H-----GDGL FAVSEIDNIW RIDPQSLDTV EKMAVSDHMA VHMATAHPLI DRNGMVY-VG  162

                           220        230        240        250        260        270        280
                    ....|....| ....|....| ....|....| ....|....| ....|....| ....|....| ....|....|
H. sapiens      194 NCFGKNFSIA YNIVKIPPLQ ADKEDPISKS EIVVQFPCSD RFKPS----- YVHSFGLTPN YIVFVETPVK  258
EVMG013855.1    183 SNY-RDYKRA FNILAFPQPA NGELDSMKTG RIVASIPSRW KFHYG----- YIHSFGMAEK YFVLLEQPCT  246
EVMG012710.1    104 SNY-RDYKRP FNILAFPQPA NGELDSMKTG RIVASIPSRW KFHCG----- YIHSFGMAER YFVLLEQPCT  167
EVMG008120.1    206 PLPEPRRPSF LTFLSEIRLL PGEKDAISLV RIHTVTRREV VARWEVDRVP YVHSFSVTEN HAVVFASPYH  275
EVMG016245.1    23  SNY-RDYKRP FNILAFPQPA NGELDSMKTG RIVASIPSRW KFHYG----- YIHSFGMAER YFVLLEQPCT  86
EVMG009860.1    185 SNY-RDYKRA FNILAFPQPA NGELDSMKTG RIVASIPSRW KFHYG----- YIHSFGMAEK YLVLLEQPCT  248
EVMG010907.1    163 SNY-RDYKRP FNILAFPQPA NGRIISTS-- -FIISTLREN KHKWR----- LINSP----K CLHCKEQ-ED  218

                           290        300        310        320        330        340        350
                    ....|....| ....|....| ....|....| ....|....| ....|....| ....|....| ....|....|
H. sapiens      259 INLFKFLSSW SLWGANYMDC FESNETMGVW LHIADKKRKK YLNNKYRTSP FN-LFHHINT YEDNGFLIVD  327
EVMG013855.1    247 YSLFKLLFRQ -VHKYSPLDA MENYENEQML FHIIRKSDGK RLSTTYKSSD VKFCFHHINT YEEEGHLVVD  315
EVMG012710.1    168 FSIFKLIFRQ -VCKYSPLDA MENYENEQML FHIIRKSDGK RLSTTYKSSD VKFCFHHINT YEEEGHLVVD  236
EVMG008120.1    276 VSVLKMVDTA -----RALDS LEWRGDSPCM IYVVDLRSGQ --VHTLKTEA MFFMHHANAF ELDDSRLVVD  338
EVMG016245.1    87  YSIFKLIFRQ -VCKYSPLDA MENYENEQML FHIIRKSDGK RLSTTYKSSD VKFCSHHINT YEEEGHLVVD  155
EVMG009860.1    249 YSIFKLIFRQ -VCKYSPLDA MENYENEQ-- ---------- ---------- ---------- ----------  275
EVMG010907.1    219 DTVLHCIF-- -ACPLN--IE MRPLVRDSML FHIIRKSDGK RLSTTYKSSD VKFCFHHINT YEEEGHLVVD  283

                           360        370        380        390        400        410        420
                    ....|....| ....|....| ....|....| ....|....| ....|....| ....|....| ....|....|
H. sapiens      328 LCCWKGFEFV YNYLYLANLR ENWEEVKKNA RKAPQPEVRR YVLPLNIDKA DTGKNLVTLP NTTATAILCS  397
EVMG013855.1    316 ICGYNDFSIV NNFRLEN--- -----LRLFS DTSTATFKRF VLPIDVTEKM PLGKNLVNLK YTTATAVKQP  377
EVMG012710.1    237 ICGYNDFNIV NNFRLEN--- -----LRLSS DTSTATFKRF VLPIDVTEKM PLGKNLVNLK DTTATAVKQP  298
EVMG008120.1    339 IAVYEDPSFI NQMTLEN--- ---------- ---------- ----LL-  D PNKRNGIDLA PQLKRYTLNI  378
EVMG016245.1    156 ICGYNDFNIV NNFRLEN--- -----LRLSS DTSTATFKRF VLPIDVTEKM PLGKNLVNLK DTTATAVKQP  217
EVMG009860.1    275 ---------V TN-------- ---------- ---------- ---------- ---------- ----------  278
EVMG010907.1    284 ICGYNDLNIV NNFRLEN--- -----LRLSS DTSTATFKRF VLPIDVTEKM PLGKNLVNLK DTTATAVKQP  345

                           430        440        450        460        470        480        490
                    ....|....| ....|....| ....|....| ....|....| ....|....| ....|....| ....|....|
H. sapiens      398 DETIWLEPEV LFSGP--RQA FEFPQINYQK YCGKPYTYAY GLG----LNH FVPDRLCKLN VKTKETWVWQ  461
EVMG013855.1    378 DGSVLLCCDN LTEDPACPIY LELPQINYEA CAGRNYRYVY GT-------- LLKTEIIKID ILMRTVNKWV  439
EVMG012710.1    299 DGSVLLCCDN LTEDPARPVY LELRQINYEA RAGRNYRYVY GT-------- LLKTEIIKID ILMRTVNKWV  360
EVMG008120.1    379 TTKAVSIQTF HTKRDRFISR LEFPTIN-ED YRARNYCYVY GFVGKMDGKR LATNALVKKD LCGNGMDRYW  447
EVMG016245.1    218 DGSVLLCCDN LTEDPARPVY LELPQINYEA RAGRNYRYVY GT-------- LLKTEIIKID ILMRTVNKWV  279
EVMG009860.1    278 ---------- -------PRQ LCVPKICLEG SV-------- ---------- ---------- ----------  293
EVMG010907.1    346 DGSVLLCCDN LTEDPARPVY LELPQINYEA RAGRNYRYVY GT-------- LLKTEVG--- --------LG  396

                           500        510        520        530        540        550        560
                    ....|....| ....|....| ....|....| ....|....| ....|....| ....|....| ....|....|
H. sapiens      462 EPD-SYPSEP IFVSHPDALE EDDGVVLSVV VSPGAGQKPA YLLILNAKDL SEVARAEVEI N--IPVTFHG  528
EVMG013855.1    440 EPNGFLAAEP VFVPRPSGED EDDGVILIPV TSSDP-ERPS YLAILDAHTL EEVAKADVPT DTFIPITFHG  508
EVMG012710.1    361 EPNGFLAAEP VFVPRPSGED EDDGVILIPV TSSDP-ERPS YLAILDAHTL EEVAKADVPT DTFIPITFHG  429
EVMG008120.1    448 SESYHYGSEL WFVPNPAGTR EDDGVLLSPV LNGTK--GQS YLAVFDARSM KLMNVG--YL PTYIPTTIHG  513
EVMG016245.1    280 EPNGFLAAEP VFVPRPSGED EDDGVILIPV TSSDP-ERPS YLAVLDAHTL E--------- ----------  329
EVMG009860.1    293 ---------- ---------- ---------- ---------- ---------- ---------- ----------  293
EVMG010907.1    397 GTKRLFAAEP VFVPRPSGED EDDGVILIPV TSSDP-ERPS YLAILDAHTL EEVAKADVPT DTFIPITFHG  465

                           570
                    ....|....| ....|
H. sapiens      529 LFKKS----- ----- 533
EVMG013855.1    509 FFDPQQQQL- ----- 517
EVMG012710.1    430 FFDPQQQQL- ----- 438
EVMG008120.1    514 RFFENI---- ----- 519
EVMG016245.1    329 ---------- ----- 329
EVMG009860.1    293 ---------- ----- 293
EVMG010907.1    466 FFDPSSSCKL WVVGE 480
```

Supplementary Fig. 14 | Sequence alignment of RPE65 of *N. pompilius* and RPE65 of *H. sapiens*. Sequence alignment was conducted and displayed using Bioedit software, between six RPE65 sequences of *N. pompilius* and one of *H. sapiens*. The conserved residues in RPE65 were marked with color background, and EVMG013855.1 retained the conserved domains as in *H. sapiens* RPE65.

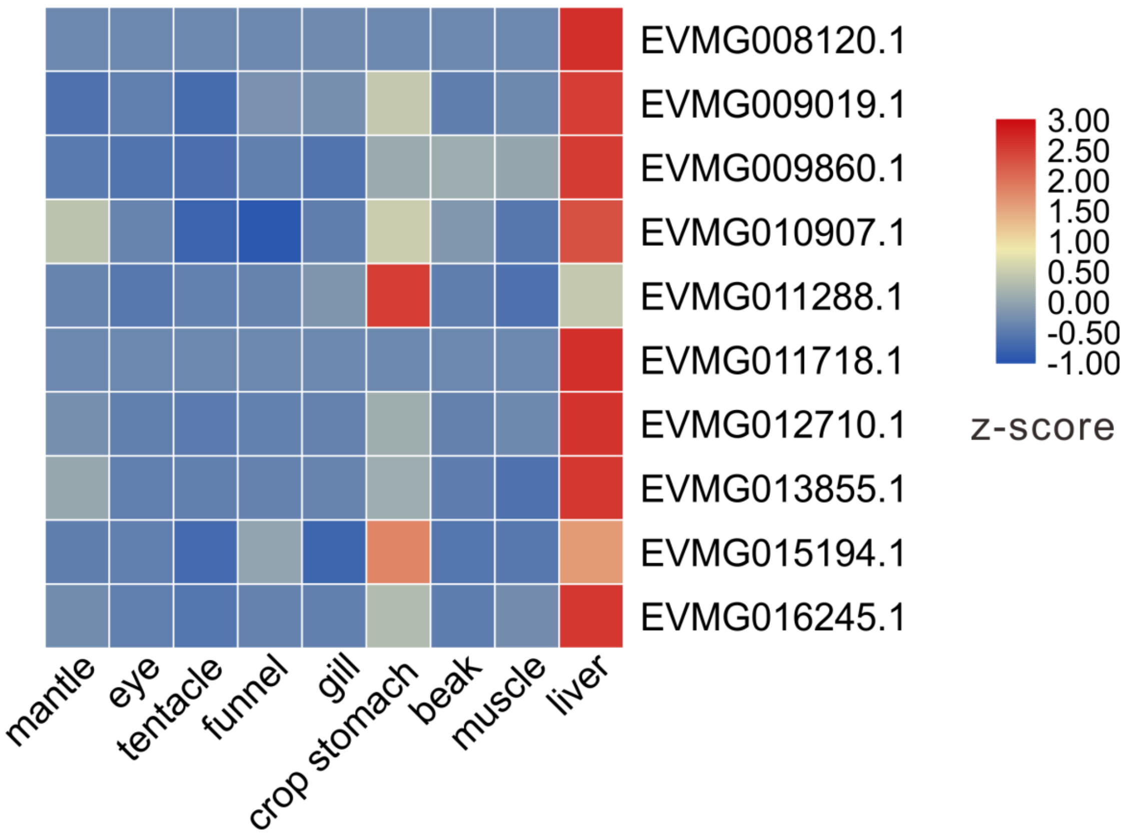

Supplementary Fig. 15 | Expression pattern of RPE65 family in *N. pompilus*. Expression level of RPE65 gene family were analyzed by using transcriptomic data in different tissues, which showed high expression of RPE65 genes in the liver.

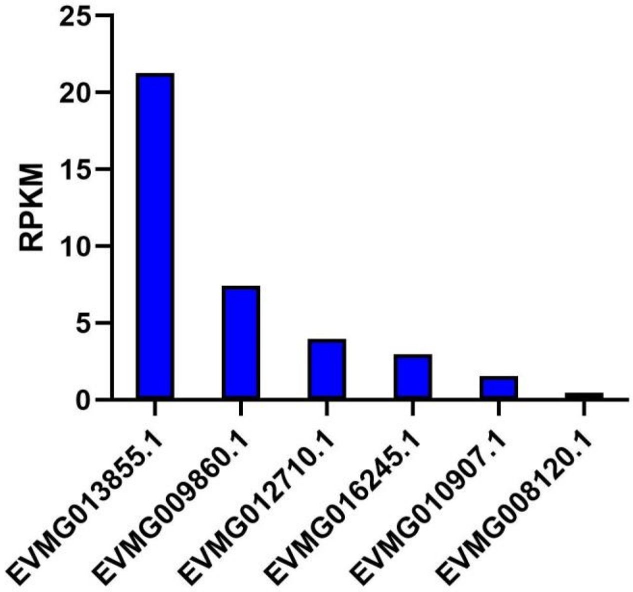

Supplementary Fig. 16 | The expression pattern of RPE65 families in the Nautilus eye. Six members of the RPE65 family genes were detected to be expressed in the eye.

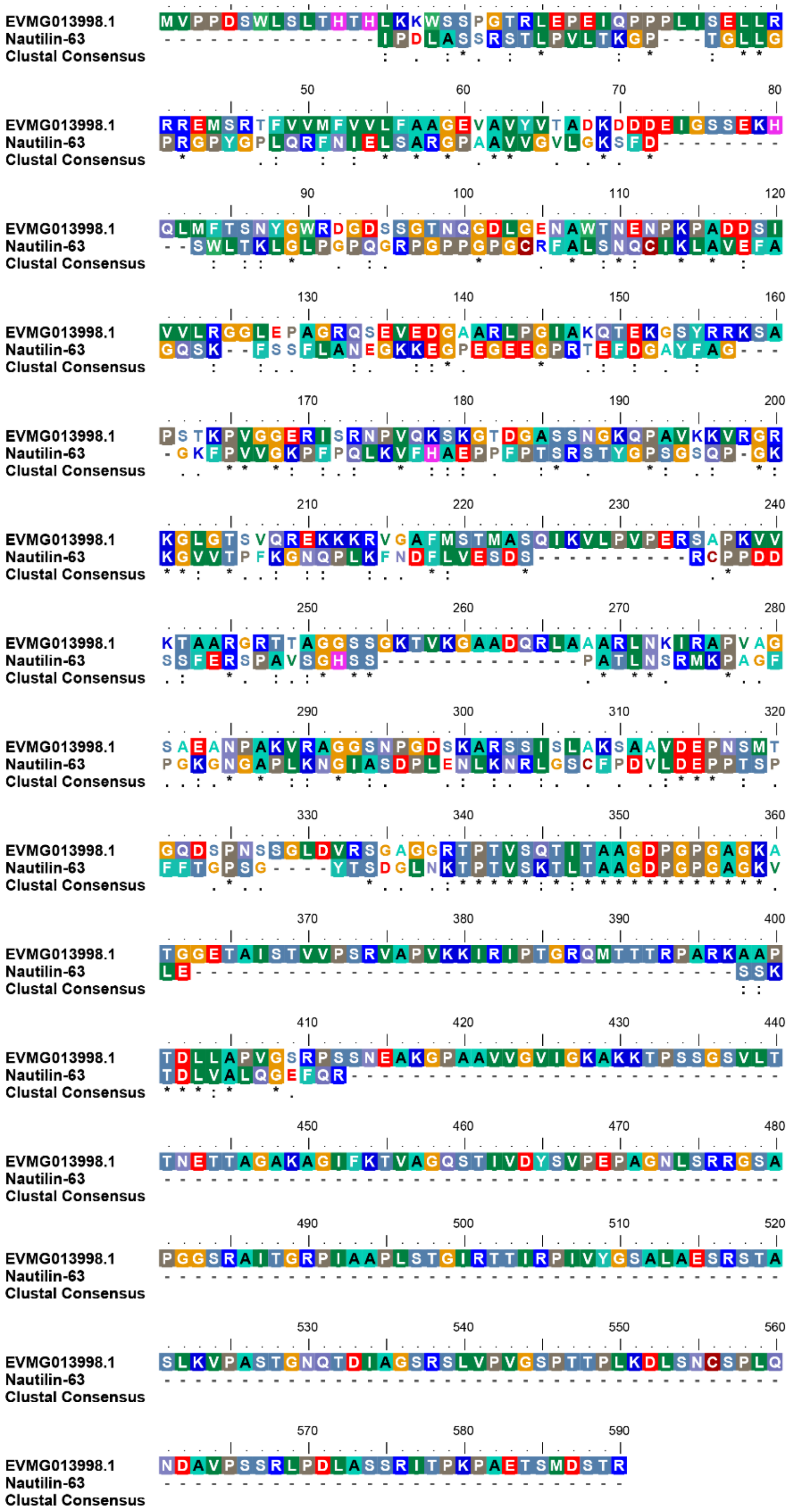

Supplementary Fig. 17 | Sequence alignment between Nautilin-63 in *Nautilus macromphalus* and EVMG013998.1 in *N. pompilius*. The identical, highly conserved, and less conserved amino acid residues are indicated by '*', ':' and '.', respectively.

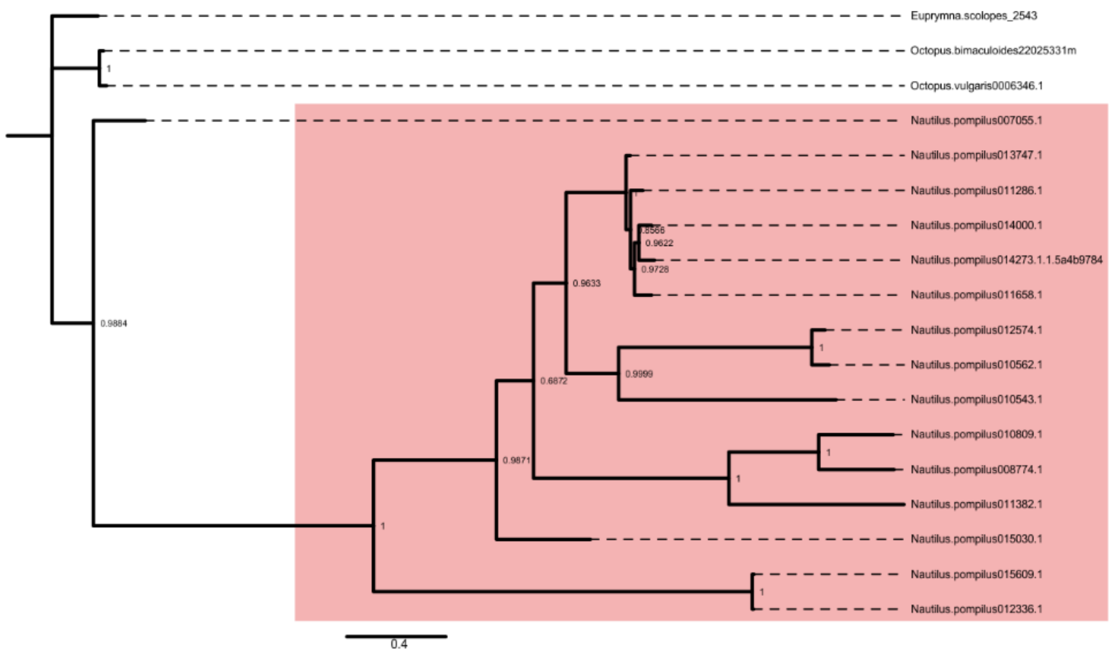

Supplementary Fig. 18 | Expansion of IFN-inducible GTPases (IIG) gene family in the *N. pompilus* genome. Phylogenetic tree of IIG proteins in cephalopods was constructed by using MrBayes methods as described above, and contains 15 of IIG proteins in *N. pompilus*, and only single IIG proteins in other cephalopods.

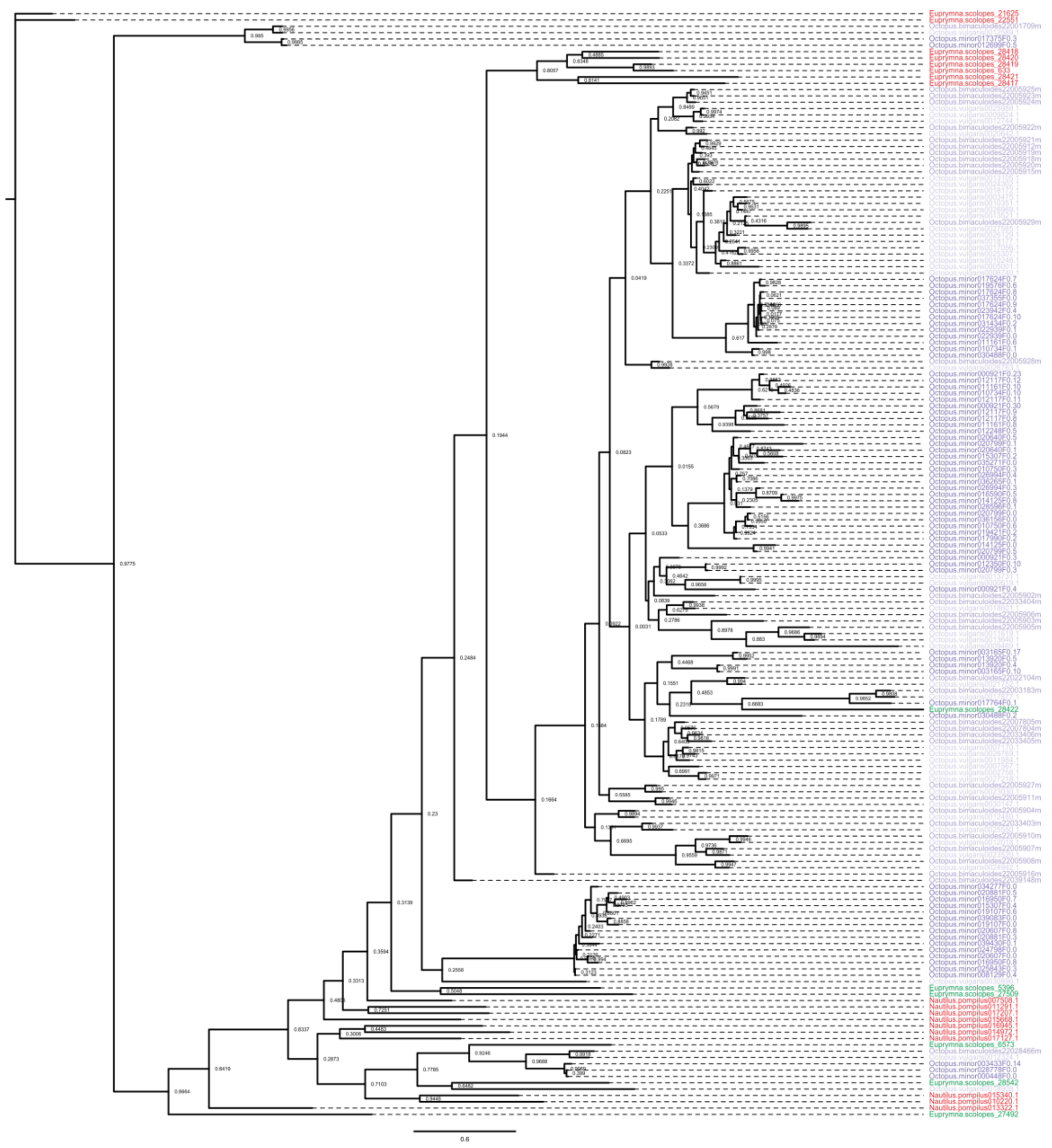

Supplementary Fig. 19 | Phylogenetic tree of interleukin-17 (IL-17) gene family in cephalopods. Phylogenetic tree of IL-17 was constructed by MrBayes method as described above, and includes 10 of IL-17 in *N. pompilus*, 14 of IL-17 in *E. scolopes*, 34 of IL-17 in *O. bimaculoides*, 72 of IL-17 in *O. minor* and 45 of IL-17 in *O. vulgaris*. Independent expansion of IL-17 gene family was found in three octopus species, strongly suggestive of a crucial role of IL-17 in octopus immune defense.

References

1. Sun, J. *et al.* Signatures of Divergence, Invasiveness, and Terrestrialization Revealed by Four Apple Snail Genomes. *Mol Biol Evol* **36**, 1507-1520 (2019).
2. Simakov, O. *et al.* Insights into bilaterian evolution from three spiralian genomes. *Nature* **493**, 526-531 (2013).
3. Zhang, G.F. *et al.* The oyster genome reveals stress adaptation and complexity of shell formation. *Nature* **490**, 49-54 (2012).
4. Sun, J. *et al.* Adaptation to deep-sea chemosynthetic environments as revealed by mussel genomes. *Nature Ecology & Evolution* **1**(2017).
5. Wang, S. *et al.* Scallop genome provides insights into evolution of bilaterian karyotype and development. *Nature Ecology & Evolution* **1**(2017).
6. Nam, B.H. *et al.* Genome sequence of pacific abalone (Haliotis discus hannai): the first draft genome in family Haliotidae. *Gigascience* **6**(2017).
7. Du, X.D. *et al.* The pearl oyster Pinctada fucata martensii genome and multi-omic analyses provide insights into biomineralization. *Gigascience* **6**(2017).
8. Albertin, C.B. *et al.* The octopus genome and the evolution of cephalopod neural and morphological novelties. *Nature* **524**, 220-4 (2015).
9. Kim, B.M. *et al.* The genome of common long-arm octopus Octopus minor. *Gigascience* **7**(2018).
10. Zarrella, I. *et al.* The survey and reference assisted assembly of the Octopus vulgaris genome. *Scientific Data* **6**(2019).
11. Belcaid, M. *et al.* Symbiotic organs shaped by distinct modes of genome evolution in cephalopods. *Proceedings of the National Academy of Sciences of the United States of America* **116**, 3030-3035 (2019).
12. Nakamura, T., Yamada, K.D., Tomii, K. & Katoh, K. Parallelization of MAFFT for large-scale multiple sequence alignments. *Bioinformatics* **34**, 2490-2492 (2018).
13. Edgar, R.C. MUSCLE: multiple sequence alignment with high accuracy and high throughput. *Nucleic Acids Research* **32**, 1792-1797 (2004).
14. Tommaso, P. *et al.* T-Coffee: a web server for the multiple sequence alignment of protein and RNA sequences using structural information and homology extension. *Nucleic Acids Research* **39**, W13-W17 (2011).
15. Lassmann, T. & Sonnhammer, E.L. Kalign, Kalignvu and Mumsa: web servers for multiple sequence alignment. *Nucleic Acids Res* **34**, W596-9 (2006).
16. Ronquist, F. *et al.* MrBayes 3.2: efficient Bayesian phylogenetic inference and model choice across a large model space. *Syst Biol* **61**, 539-42 (2012).
17. Letunic, I., Doerks, T. & Bork, P. SMART: recent updates, new developments and status in 2015. *Nucleic Acids Res* **43**, D257-60 (2015).
18. Bailey, T.L. & Machanick, P. Inferring direct DNA binding from ChIP-seq. *Nucleic Acids Res* **40**, e128 (2012).
