# Supplementary material for: The genome of Nautilus pompilius illuminates eye evolution and biomineralization
Source: Nat Ecol Evol. 2021 May 10;5(7):927–38. doi: 10.1038/s41559-021-01448-6 (PMC8257504; doi:10.1038/s41559-021-01448-6)
Supplement: Supplementary file 2 — Reporting Summary [file 41559_2021_1448_MOESM2_ESM.pdf]

nature research

Corresponding author(s): Ziniu Yu

Last updated by author(s): May 29, 2020

Reporting Summary

Nature Research wishes to improve the reproducibility of the work that we publish. This form provides structure for consistency and transparency in reporting. For further information on Nature Research policies, see our Editorial Policies and the Editorial Policy Checklist.

Statistics

For all statistical analyses, confirm that the following items are present in the figure legend, table legend, main text, or Methods section.

n/a	Confirmed	
☒	☐	The exact sample size (*n*) for each experimental group/condition, given as a discrete number and unit of measurement
☒	☐	A statement on whether measurements were taken from distinct samples or whether the same sample was measured repeatedly
☒	☐	The statistical test(s) used AND whether they are one- or two-sided<br>*Only common tests should be described solely by name; describe more complex techniques in the Methods section.*
☒	☐	A description of all covariates tested
☒	☐	A description of any assumptions or corrections, such as tests of normality and adjustment for multiple comparisons
☒	☐	A full description of the statistical parameters including central tendency (e.g. means) or other basic estimates (e.g. regression coefficient) AND variation (e.g. standard deviation) or associated estimates of uncertainty (e.g. confidence intervals)
☒	☐	For null hypothesis testing, the test statistic (e.g. *F*, *t*, *r*) with confidence intervals, effect sizes, degrees of freedom and *P* value noted<br>*Give P values as exact values whenever suitable.*
☒	☐	For Bayesian analysis, information on the choice of priors and Markov chain Monte Carlo settings
☒	☐	For hierarchical and complex designs, identification of the appropriate level for tests and full reporting of outcomes
☒	☐	Estimates of effect sizes (e.g. Cohen's *d*, Pearson's *r*), indicating how they were calculated

Our web collection on statistics for biologists contains articles on many of the points above.

Software and code

Policy information about availability of computer code

Data collection	llumina Hiseq 2500 platform, BWA v0.7.13, FastUniq v1.1, g-TUBE device, PacBio RS-II platform.
Data analysis	Canu v1.5, MHAP v2.12, LoRDEC v0.6, WTDBG 1.1.006, Sparse software, Sparc module and blasr software, MUMmer v4.0.0, quickmerge, Pilon v1.22, BUSCO v3.0.2b, LTR-FINDER, MITE-Hunter, RepeatScout, PILER-DF, PASTEClassifier, Repbase database, RepeatMasker program,GeMoMa v1.3.1, Stringtie v1.2.3, PASA, TransDecoder v2.0, GeneMarkS-T v5.1, Genscan, Augustus v2.4, GlimmerHMM v3.0.4, GeneID v1.4, SNAP v2006-07-28, Trinity vTrinityrnaseq_r20131110, COG, KEGG, NCBI-NR, Swiss-Prot, MUSCLE v3.8.31, RAxML v8.2.12, PAML package, CAFE, InParanoid v4.1 software, MUSCLE v3.8.31, MEGA v7.0.18, PSMC v0.6.5 software, TopHat v2.1.1, Cufflinks v2.1.1

For manuscripts utilizing custom algorithms or software that are central to the research but not yet described in published literature, software must be made available to editors and reviewers. We strongly encourage code deposition in a community repository (e.g. GitHub). See the Nature Research guidelines for submitting code & software for further information.

Data

Policy information about availability of data

All manuscripts must include a data availability statement. This statement should provide the following information, where applicable:

- Accession codes, unique identifiers, or web links for publicly available datasets
- A list of figures that have associated raw data
- A description of any restrictions on data availability

The nautilus genome project has been deposited at NCBI under the BioProject number PRJNA614552. The WGS data was deposited in the Sequence Read Archive (SRA) database under the accession numbers SRR11485669- SRR11485706. RNA-seq data of various tissue transcriptomes have also been deposited in the SRA database under the accession numbers SRR11485678- SRR11485687.

Field-specific reporting

Please select the one below that is the best fit for your research. If you are not sure, read the appropriate sections before making your selection.

☒ Life sciences ☐ Behavioural & social sciences ☐ Ecological, evolutionary & environmental sciences

For a reference copy of the document with all sections, see nature.com/documents/nr-reporting-summary-flat.pdf

Life sciences study design

All studies must disclose on these points even when the disclosure is negative.

Sample size	single individual
Data exclusions	N/A
Replication	N/A
Randomization	N/A
Blinding	N/A

Reporting for specific materials, systems and methods

We require information from authors about some types of materials, experimental systems and methods used in many studies. Here, indicate whether each material, system or method listed is relevant to your study. If you are not sure if a list item applies to your research, read the appropriate section before selecting a response.

Materials & experimental systems

n/a	Involved in the study	
☒	☐	Antibodies
☒	☐	Eukaryotic cell lines
☒	☐	Palaeontology and archaeology
☐	☒	Animals and other organisms
☒	☐	Human research participants
☒	☐	Clinical data
☒	☐	Dual use research of concern

Methods

n/a	Involved in the study	
☒	☐	ChIP-seq
☒	☐	Flow cytometry
☒	☐	MRI-based neuroimaging

Animals and other organisms

Policy information about studies involving animals; ARRIVE guidelines recommended for reporting animal research

Laboratory animals	N/A
Wild animals	One single Nautilus pompilius donated by the Chinese Ocean Conservation Association was used in the study in accordance with local guidelines and regulations on animal experiments. All experimental protocols had been reviewed and approved by the research ethics committee for animal experiments at the South China Sea Institute of Oceanology, Chinese Academy of Sciences.
Field-collected samples	N/A
Ethics oversight	N/A

Note that full information on the approval of the study protocol must also be provided in the manuscript.
